# The Interplay of NEAT1 and miR-339-5p Influences on Mesangial Gene Expression and Function in Various Diabetic-Associated Injury Models

**DOI:** 10.3390/ncrna8040052

**Published:** 2022-07-13

**Authors:** Simone Reichelt-Wurm, Matthias Pregler, Tobias Wirtz, Markus Kretz, Kathrin Holler, Bernhard Banas, Miriam C. Banas

**Affiliations:** 1 Department of Nephrology, University Hospital Regensburg, 93053 Regensburg, Germany; mpregler94@gmx.de (M.P.); tobias.wirtz@ukr.de (T.W.); kathrin.holler@ukr.de (K.H.); bernhard.banas@ukr.de (B.B.); miriam.banas@ukr.de (M.C.B.); 2 Regensburg Center for Biochemistry (RCB), University of Regensburg, 93053 Regensburg, Germany; markus.kretz@ur.de

**Keywords:** *NEAT1*, has-miR-339-5p, diabetic kidney disease, mesangial cell, lncRNA, miRNA

## Abstract

Mesangial cells (MCs), substantial cells for architecture and function of the glomerular tuft, take a key role in progression of diabetic kidney disease (DKD). Despite long standing researches and the need for novel therapies, the underlying regulatory mechanisms in MCs are elusive. This applies in particular to long non-coding RNAs (lncRNA) but also microRNAs (miRNAs). In this study, we investigated the expression of nuclear paraspeckle assembly transcript 1 (*NEAT1*), a highly conserved lncRNA, in several diabetes in-vitro models using human MCs. These cells were treated with high glucose, TGFβ, TNAα, thapsigargin, or tunicamycin. We analyzed the implication of *NEAT1* silencing on mesangial cell migration, proliferation, and cell size as well as on mRNA and miRNA expression. Here, the miRNA hsa-miR-339-5p was not only identified as a potential interaction partner for *NEAT1* but also for several coding genes. Furthermore, overexpression of hsa-miR-339-5p leads to a MC phenotype comparable to a *NEAT1* knockdown. In-silico analyses also underline a relevant role of *NEAT1* and hsa-miR-339-5p in mesangial physiology, especially in the context of DKD.

## 1. Introduction

With its latest report, the International Diabetes Federation estimated diabetes prevalence at 463 million people for 2019 and prognosticates an increase to 700 million by 2045. An estimated 90% of these individuals are affected by type 2 diabetes mellitus (T2DM) [1]. Approximately 40% of T2DM patients develop diabetic kidney disease (DKD), which represents the leading cause of end-stage renal disease (ESRD) with the ultimate requirement of renal replacement procedures [2]. Diabetes-mediated chronic hyperglycemia induces hyperfiltration [3] as well as the onset and development of renal injury, including chronic inflammation [4], podocyte foot process effacement and detachment leading to albuminuria [3,5,6], extracellular matrix (ECM) expansion, glomerular sclerosis [7], and a declining glomerular filtration rate (GFR) [8]. 

In brief, mesangial cells (MCs) are essential for maintenance of structure, function, and integrity of the glomerular capillary tuft and are in continuous cross-talk with adjacent podocytes and endothelial cells. Besides, they share characteristics with smooth muscle cells (SMCs) [9]. T2DM and recurring hyperglycemia, however, induce diabetic injury in MCs comprising hypertrophy [10], ECM protein synthesis [11], and endoplasmic reticulum (ER) stress [12]. Strikingly, T2DM can result in both proliferation and ECM accumulation [11] and, in contrast, in apoptosis and mesangiolysis [13,14]. But the underlying mechanisms, which prescribe the direction, remain elusive [15].

More than 95% of the transcriptome is non-coding, yet with structural or functional relevance [16,17]. MicroRNAs (miRNAs) and long non-coding RNAs (lncRNAs) represent two important classes of non-coding RNAs (ncRNAs) with regulatory character. After processing by Microprocessor and Dicer enzyme, miRNAs act as single-stranded transcripts with an average length of 22 nucleotides, which derive their effect by association with an Argonaut protein [18,19]. In this complex, miRNAs bind to complementary sequences of their target messenger RNAs (mRNAs) and mediate either degradation or translational repression of the mRNA [19]. In contrast, lncRNAs are longer than 200 nucleotides and have a far broader spectrum of activity, but lack protein-coding capacity. They can affect virtually every step of gene expression including pre- and post-transcriptional and -translational control, splicing, and genomic imprinting [20,21]. Research has revealed their relevance in development, cancer, and various diseases, like DKD [22,23] and renal fibrosis [24]. 

A single lncRNA can be involved in several mechanisms, as in the case of nuclear paraspeckle assembly transcript 1 (*NEAT1*). This transcript is a highly conserved lncRNA occurring in two isoforms with the same 5′-end. In humans, the short variant (3684 nucleotides), referred as *NEAT1_1*, is produced by early 3′-end processing while the long *NEAT1*_2 isoform (22743 nucleotides) is formed without poly(A) tail by RNase P cleavage at a tRNA-like structure [25,26]. As a structural element, *NEAT1* comprises a core component of nuclear paraspeckles [27]; nevertheless, it also acts as competing endogenous RNA (ceRNA) [28,29,30,31,32] to de-repress [33] gene expression by competing with miRNAs for interaction with shared target mRNAs. 

In the present study, we aimed to analyze the role of *NEAT1* in human MCs (hMCs) in various in-vitro diabetes models. Thereby, we focused on *NEAT1*’s role as ceRNA and identified the miRNA hsa-miR-339-5p (miR-339-5p) as a potential interaction partner, not only for *NEAT1* but also for various coding genes, which play highly relevant roles in MCs during development and progression of DKD.

## 2. Results

### 2.1. NEAT1 Expression in Human Mesangial Cells

**Figure 1:**
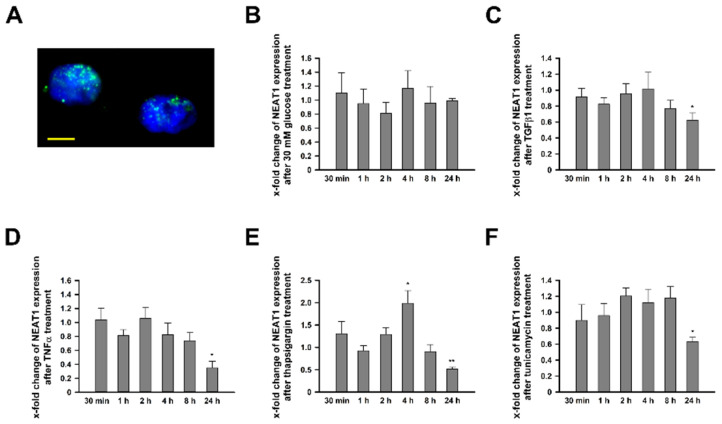
*NEAT1_1*/2 expression in human mesangial cell (hMCs) under basal conditions and after stimulation. (**A**) In-situ detection of *NEAT1_1/2* (green) in untreated cultured hMCs. The nuclei are stained blue by DAPI. The scale bar indicates 5 µm. (**B**–**F**) Fold change of *NEAT1_1/2* (grey bars) expression in hMCs after stimulation with (**B**) 30 mM glucose normalized to mannitol, (**C**) TGFβ1 normalized to medium, (**D**) TNFα normalized to medium, (**E**) thapsigargin normalized to DMSO, and (**F**) tunicamycin normalized to DMSO. Bars represent x-fold changes + SD. Overall significance of differences was analyzed by ANOVA, followed by Student’s *t*-tests for post hoc pairwise comparisons. * *p* < 0.05; ** *p* < 0.001 compared to corresponding control treatment; *n* = 3–4.

*NEAT1* expression was ascertained by quantitative polymerase chain reaction (qPCR) and in-situ hybridization (ISH) analysis. In unstimulated hMCs, we ascertained cycle threshold (CT) values of 22.98 for *NEAT1_1*/2 and 23.96 for *NEAT1_2*. Thus, *NEAT1* represents a gene with moderate to high expression level. Counting back to the expression of *NEAT1_1*, we observed that both variants are equally strong expressed (data not shown). Detection via ISH confirmed the nuclear location with the typical inhomogeneous distribution and concentration in foci (Figure 1A). Next, we wanted to analyze alterations of *NEAT1* expression in various in-vitro T2DM models. Besides high glucose (HG) treatment, hMCs were also stimulated with tunicamycin (TM) and thapsigargin (TG) as well as transforming growth factor β1 (TGFβ1) and tumor necrosis factor α (TNFα) to mimic diabetes evoked endoplasmic reticulum (ER) stress or the increased release of profibrotic or proinflammatory molecules to address multiple aspects of DKD [34]. While hMCs under hyperglycemic conditions did not exhibit changes in *NEAT1* expression, all other treatments caused a significantly reduced *NEAT1* expression after 24 h (Figure 1B–F). Noteworthy, short term exposure of 4 h to TG resulted in a transient but significant increase in expression. A more differentiated analysis, distinguishing between *NEAT1_1* and *NEAT1_2*, revealed no considerable difference between these transcript variants (Supplementary Figure S1).

### 2.2. NEAT1 Knockdown in Human Mesangial Cells Affects Proliferation, Cell Size, and Migration

**Figure 2:**
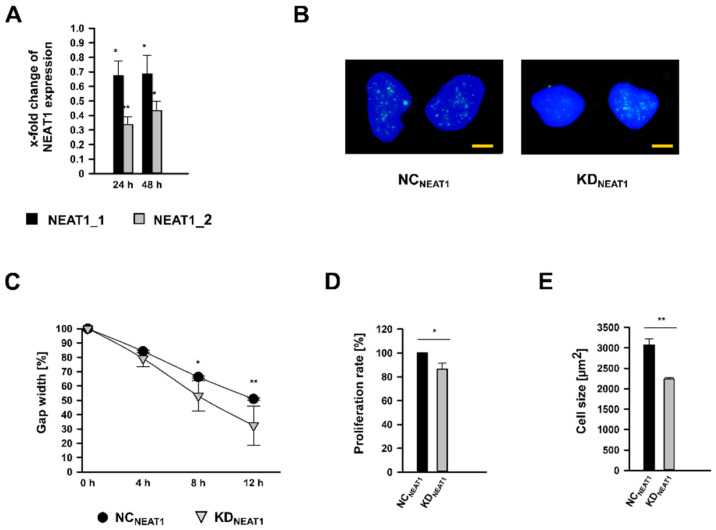
*NEAT1* silencing in human mesangial cells (hMCs) using siPools targeting both *NEAT1_1* and *NEAT1_2*. (**A**) Efficiencies of *NEAT1* knockdown (KD*_NEAT1_*) after 24 or 48 h shown as x-fold changes + SD for *NEAT1_1* (black bars) and *NEAT1_2* (grey bars) RNA expression normalized to the expression after treatment with scrambled negative control (NC*_NEAT1_*) siRNA; *n* = 3. (**B**) In-situ detection of *NEAT1_1/2* (green) in NC*_NEAT1_* and KD*_NEAT1_* treated hMCs. The nuclei are stained blue by DAPI. The scale bar indicates 5 µm. (**C**) Cell migration was analyzed by wound healing assay by measuring the gap width, directly after removing the cell culture insert (0 h) and 4 h, 8 h, and 12 h later. Black circles ± SD or grey triangles ± SD represent NC*_NEAT1_* and KD*_NEAT1_* treated hMCs, respectively; *n* = 3. (**D**) Proliferation rate of hMCs after KD*_NEAT1_* (grey bar + SD) compared to NC*_NEAT1_* (black bar). Proliferation was ascertained by a BrdU assay; *n* = 5. (**E**) Cell size in µm^2^ of hMCs after KD*_NEAT1_* (grey bar + SD) compared to NC*_NEAT1_* (black bar + SD). Cell size measurement based on WGA immunostaining followed by morphometric analyses; *n* = 3. Overall significance of differences was analyzed by ANOVA, followed by Student’s *t*-tests for post hoc pairwise comparisons. * *p* < 0.05; ** *p* < 0.001.

Both *NEAT1* transcripts were silenced using siPOOLs (KD*_NEAT1_*; KD: knockdown), which bind exclusively within the overlapping 5-end, whereby both isoforms could be targeted. RNA expression analysis after 24 and 48 h of treatment revealed that both transcripts were significantly less expressed in KD*_NEAT1_* hMCs compared to cells subjected to scrambled siPOOLs, which served as negative control (NC*_NEAT1_*; NC: negative control). Surprisingly, *NEAT1* siPOOLs affected *NEAT1_2* with more enhancement at both times (Figure 2A). The reduced amount of *NEAT1* RNA after KD*_NEAT1_* could also be confirmed by ISH. The KD*_NEAT1_* does not seem to influence the nuclear expression pattern of the lncRNA (Figure 2B).

The reduced expression of *NEAT1* affected hMC physiology. Migratory capabilities were examined by a wound-healing assay. After 8 h, the gap width after KD*_NEAT1_* was already significantly diminished by approximately 50%. A further 4 h later, the gap was closed to two-thirds, in contrast to NC*_NEAT1_* cells, where the space was only half covered with hMCs (Figure 2C and Figure S2). After 24 h, both gaps were completely closed. Conducting a BrdU proliferation assay, we observed a significant decrease of cell replication by 13% (Figure 2D). Furthermore, morphometric cell size analysis revealed that hMCs were 28% smaller than control cells (Figure 2E). The reduced cell size after KD*_NEAT1_* is also supported by the ISH experiment (Figure 2B).

### 2.3. Analysis of Differentially Expressed Coding Genes after NEAT1 Knockdown

**Figure 3:**
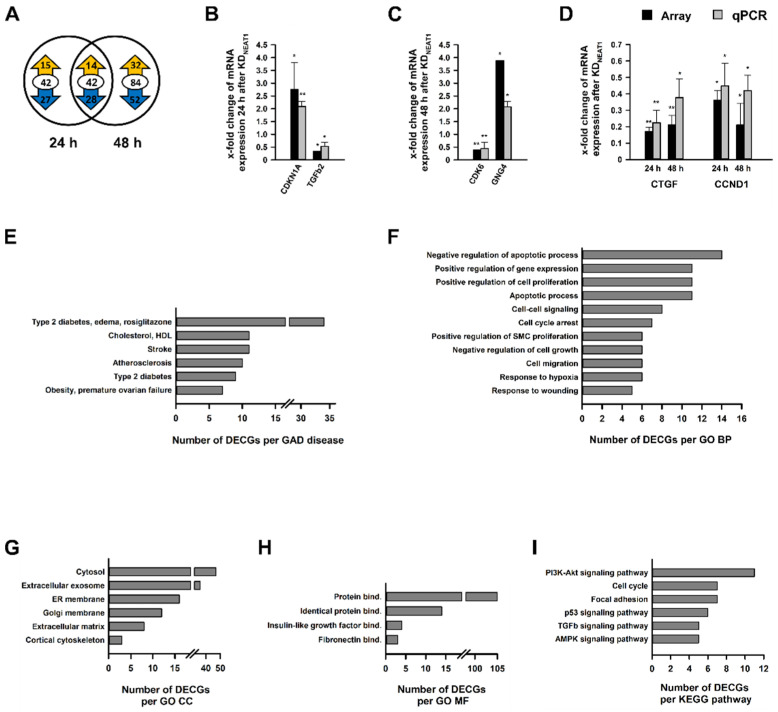
Analysis of differentially expressed coding genes (DECGs) in human mesangial cells (hMCs) after *NEAT1* silencing compared to negative control siRNA treated cells. (**A**) The Venn diagram depicts the number of DECGs 24 h (left) and 48 h (right) after the *NEAT1* knockdown in hMCs. The overlapping region between both circles represents genes which were differentially expressed at both times. The encircled value corresponds to the total number of DECGs, and the cyphers in the yellow or blue arrow represent the number of up- and downregulated DECGs, respectively; *n* = 3. (**B**–**D**) Validation of selected DECGs found in the microarray via qPCR, with (**B**) DECGs (*CDKN1A* and TGFb2) after 24 h, (**C**) DECGs (*CDK6* and *GNG4*) after 48 h, and (**D**) DECGs (*CTGF* and *CCND1*) after 24 h and 48 h. Black bars + SD show x-fold changes of gene expression in microarray (normalized to internal controls), grey bars + SD show x-fold changes of gene expression validated by qPCR (normalized to peptidylprolyl isomerase B); * *p* < 0.05; ** *p* < 0.001; *n* = 3–4. (**E**–**I**) In-silico enrichment analyses based on all DECGs detected by microarray analysis. Illustrations show selected significantly enriched terms (*p* < 0.05; EASE score < 0.05), which are relevant for mesangial physiology, sorted by count of DECGs. The complete list is shown in the Supplement. (**E**) Functional enrichment analysis in terms of selected Genetic Association Database (GAD) diseases. (**F**–**H**) Functional enrichment analysis in terms of Gene Ontology (**GO**) with the aspects (**F**) Biological Processes (BP), (**G**) Cellular Component (CC), and (**H**) Molecular Function (MF). (**I**) Functional enrichment analysis in terms of Kyoto Encyclopedia of Genes and Genomes (KEGG) pathways. Abbreviations: HDL: high density lipoprotein; SMC: smooth muscle cell; ER: endoplasmic reticulum; bind.: binding.

After demonstrating that *NEAT1* silencing influenced hMCs’ physiology, our next aim was to identify differentially expressed coding genes (DECGs), which might play a role in these processes. Total RNA, isolated 24 h or 48 h after KD*_NEAT1_*, served as basic material for Clariom S microarray analysis. Altogether, we identified 168 DECGs (Supplementary Tables S1 and S2); 42 of these were represented in both groups. Approximately two-thirds of DECGs exhibited a reduced expression (Figure 3A). The differential expression of randomly selected DECGs was confirmed by qPCR. We validated expression of cyclin dependent kinase inhibitor 1A (*CDKN1A*) and *TGFβ2* as examples for up- and downregulated DECGs after 24 h (Figure 3B) as well as cyclin dependent kinase 6 (*CDK6*) and guanine nucleotide binding protein gamma 4 (*GNG4*) (Figure 3C) as DECGs after 48 h of NEAT silencing. Connective tissue growth factor (*CTGF*) and Cyclin D1 (*CCND1*) were differentially expressed after both 24 h and 48 h (Figure 3D). 

In-silico studies in terms of Genetic Association Database (GAD) disease analysis, Gene Ontology (GO) categories with the aspects Biological Process (BP), Cellular Component (CC), and Molecular Function (MF), as well as Kyoto Encyclopedia of Genes and Genomes (KEGG) pathways revealed a highly significant clustering and enrichment of DECGs. Figure 3E–I provides an overview of selected significantly enriched terms associated with diabetes or mesangial cells’ physiology sorted by count. Overall lists, including *p* values and genes, are shown in Supplementary Tables S3–S7. GAD disease analysis unveiled a direct link to 27 diseases, including diabetes (Figure 3E) and kidney dysfunction (Supplementary Table S3). Examples of significantly enriched GO BPs were cell migration (Figure 3F), and regulation of glucose metabolic process (Supplementary Table S4). Silencing *NEAT1* led to an enrichment of DECGs, whose place of action is located in the ER membrane or the extracellular matrix (ECM) (Figure 3G). At molecular level, a disproportionately number of DECGs are involved in protein binding; however, insulin-like growth factor and fibronectin binding were also identified (Figure 3H). Enriched KEGG pathways were cell cycle, focal adhesion, and various signaling pathways, which are important for the development of diabetes (e.g., p53 and TGFβ) (Figure 3I).

### 2.4. Analysis of Differentially Expressed miRNAs after NEAT1 Knockdown

**Figure 4:**
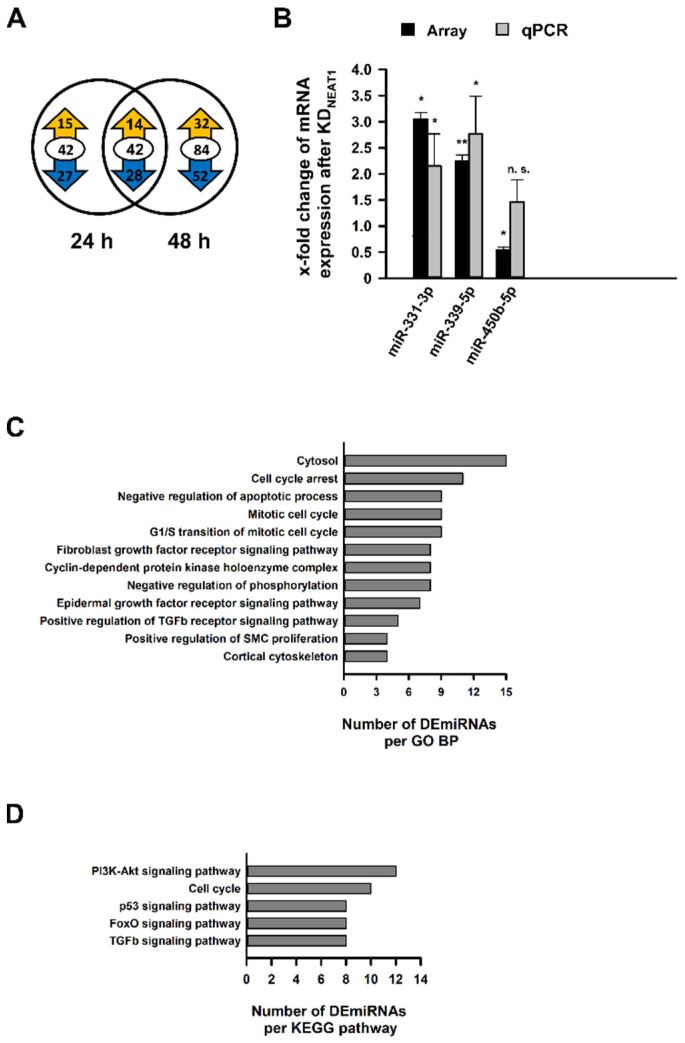
Analysis of differentially expressed miRNAs (DEmiRNAs) in human mesangial cells (hMCs) after *NEAT1* silencing compared to negative control siRNA treated cells. (**A**) The Venn diagram indicates the number of DEmiRNAs 24 h (left) and 48 h (right) after the second *NEAT1* knockdown in hMCs. The overlapping region between both circles represents miRNAs, which were differentially expressed at both times. The encircled value corresponds to the total number of DEmiRNAs, and the ciphers in the yellow or blue arrow represent the number of up- and downregulated DEmiRNAs, respectively; *n* = 3. (**B**) Validation of selected DEmiRNAs (miR-331-3p, miR-339-5p, miR-450b-5p) found in the microarray via qPCR. Black bars + SD show x-fold changes of gene expression in microarray (normalized to internal controls), grey bars + SD show x-fold changes of gene expression validated by qPCR (normalized to hsa-5S-rRNA); * *p* < 0.05; ** *p* < 0.001; n.s.: not significant; *n* = 3. (**C**,**D**) In-silico enrichment analyses based on DEmiRNAs, using DECGs detected by the microarray as filter. Illustrations show selected significantly enriched terms (*p* < 0.05; EASE score < 0.05), which are relevant for mesangial physiology, sorted by count. The complete list is shown in the Supplement. (**C**) Functional enrichment analysis in terms of Gene Ontology (GO) Biological Processes (BP). (**D**) Functional enrichment analysis in terms of Kyoto Encyclopedia of Genes and Genomes (KEGG) pathways. Abbreviations: TGFb: transforming growth factor b; SMC: smooth muscle cell; PI3K-Akt: phosphatidylinositol 3-kinase—protein kinase B; FOXO: forkhead box O.

Simultaneously to the microarray for DECGs, miRNA expression was analyzed in an identical setup using the GeneChip miRNA 4.0 Array. We identified 19 and 12 differentially expressed miRNAs (DEmiRNAs), respectively, 24 or 48 h after KD*_NEAT1_* (Supplementary Tables S8 and S9). Only two miRNAs were represented in both groups. Contrarily to DECGs, approximately two-thirds of the miRNAs exhibited an increased expression (Figure 4A). The differential expression of randomly selected DEmiRNAs was validated by qPCR (Figure 4B). For functional enrichment analysis of miRNAs, we used all DECGs as filter. Significantly enriched GO BPs led to positive regulation of SMC proliferation and regulation of cell growth (Figure 4C). We identified p53 and TGFβ1 signaling pathways as enriched KEGG pathways (Figure 4D). Figure 4C,D provides an overview of significantly enriched terms associated with diabetes or MCs’ physiology sorted by count. Overall lists, including *p* values as well as number of miRNAs and genes, are shown in Supplementary Tables S10 and S11.

### 2.5. Identification and In Silico-Characterization of the NEAT1 Binding miRNA miR-339-5p

The *NEAT1* sequence was analyzed for targeting miRNAs using the online databases RNA22, mirDIP, and Starbase. Hundreds of miRNAs can bind to both *NEAT1_1* and *NEAT1_2*; most of them have several target sites (not shown). For subsequent investigations, we excluded transcripts which were not expressed (according to mean channel intensity below 10) or not differentially expressed in our array. Three miRNAs were identified by all three databases: hsa-miR-339-5p (miR-339-5p), hsa-miR-615-3p, and hsa-miR-3180-3p. The complete list is displayed in Supplementary Table S12 (Supplementary Tables S8 and S9 show all DEmiRNAs including x fold change (xFC) and *p* value). 

Out of these, miR-339-5p seemed to be the most promising candidate for future investigations because of the highest xFC of 2.24 and the lowest *p* value of *p* = 0.0009—compared to xFC = 1.71 and *p* = 0.0160 for hsa-miR-615-3p or xFC = 1.70 and *p* = 0.0304 for hsa-miR-3180-3p. Furthermore, the significant increase in expression was validated by qPCR, providing a xFC = 2.46 and *p* = 0.031 (Figure 4B). 

**Figure 5:**
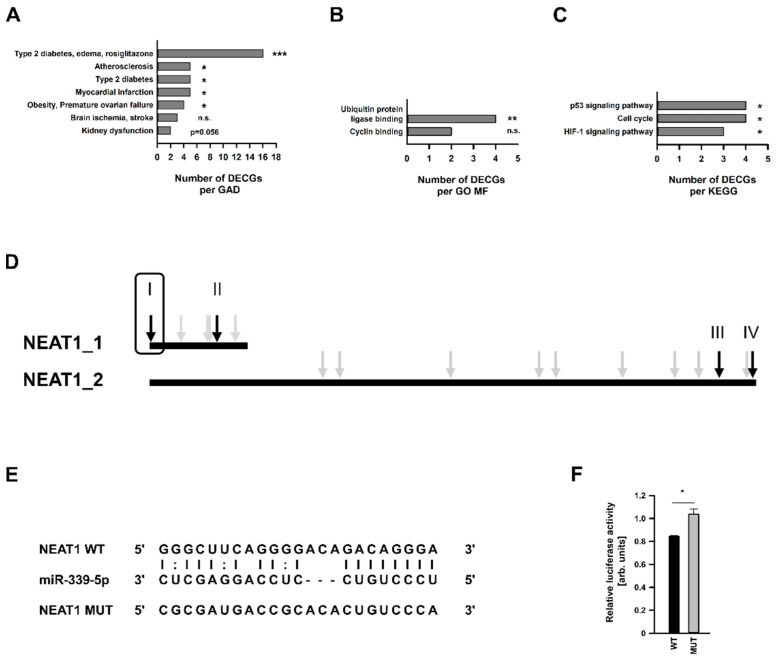
Functional enrichment analysis of differentially expressed coding genes (DECGs) in human mesangial cells (hMCs) with predicted miR-339-5p targeting site. Significant enriched terms in respect of (**A**) Genetic Association Database (GAD) diseases (the complete list is shown in the Supplement), as well as (**B**) Molecular Function (MF). (**C**) Functional enrichment analysis in terms of Kyoto Encyclopedia of Genes and Genomes (KEGG) pathways. Abbreviation: HIF: hypoxia induced factor. Selected significant terms (according to DAVID integrated algorithms) are sorted by count. Additionally, significance regarding actual over-representation of a particular term was analyzed by hypergeometric testing. * *p* < 0.05; ** *p* < 0.01; *** *p* < 0.001; n.s. = not significant. (**D**) Predicted miR-399-5p targeting sites for *NEAT1_1/2* indicated by grey and black arrows. Loci marked with black arrows were analyzed via luciferase reporter gene assays. Information and result for the site with the framed black arrow are shown in (**E**,**F**) in this figure. (**E**) Potential interaction site of miR-339-5p and *NEAT1*. The sequences of miR-339-5p, wildtype (WT) *NEAT1* and designed mutated (MUT) *NEAT1* are shown. (**F**) Dual-luciferase reporter assay in hMCs showing the effect of miR-339-5p cotransfected with pmirGlo vector containing either WT or MUT *NEAT1* sequence, displayed by black bar + SD or grey bars + SD, respectively, *n* = 3–4. DECG (grey bars). Significance was analyzed by Student’s *t*-test. * *p* < 0.05.

An additional miRNA target analysis, using the databases mentioned above, revealed that this miRNA may also interfere with a 40 further DECGs from our array (Table 1). While miR-339-5p was 2.24-fold more highly expressed after KD*_NEAT1_*, the majority of these DEGCs were downregulated. Conducting in-silico analyses using only these genes, we ascertained a clear correlation to mesangial physiology in the context of diabetes. Regarding GAD diseases, we found these genes overrepresented in the terms T2DM and kidney dysfunction (Figure 5A and further: Supplementary Table S13). Two GO MFs were also found: ubiquitin protein ligase binding and cyclin binding (Figure 5B). Moreover, we identified the p53 signaling pathway, cell cycle, and HIF1 signaling pathway as significantly enriched KEGG pathways (Figure 5C). All terms stated were significantly enriched based on the algorithms used by the Database for Annotation, Visualization, and Integrated Discovery (DAVID). Additionally, we performed hypergeometric testing to calculate a *p* value for each term which will allow a determination if this term is actually over-represented. Using only DAVID, we also identified categories of GO BPs and GO CCs, respectively (Supplementary Tables S14 and S15).

*NEAT1* was examined for miR-339-5p targeting sites using the databases RNA22 and Starbase. As shown in Figure 5D, both *NEAT1* transcripts carry several binding sites for this miRNA. We decided to examine two sites for each transcript variant in accordance with the lowest binding energy indicated (data not shown). Luciferase-based reporter gene assay was conducted as described by Campos–Melo to exclude cellular or exogenous miRNA effects regulating the luciferase activity [35]. Luciferase activity of pmirGlo vector transfected hMCs, carrying either the wildtype (WT) or mutated (MUT) target sequence (Figure 5E) in its 3′-UTR, was analyzed in three independent experiments. Only one predicted binding site at the very beginning of the 5′-end of *NEAT1* seemed to be targeted by miR-339-5p (Figure 5F), showing a significant difference of luciferase activity between the vector with WT or MUT sequence.

### 2.6. Treatment of hMCs with miR-339-5p Mimics

**Figure 6:**
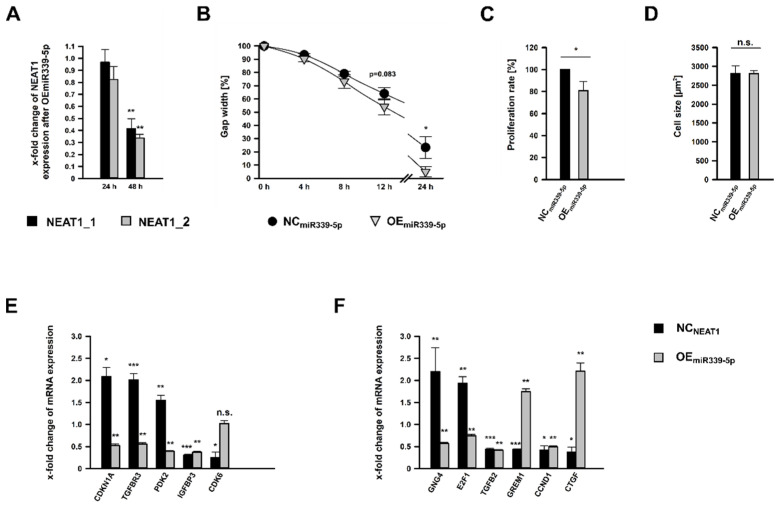
Effects of miR-399-5p overexpression (OE_miR339-5p_) on human mesangial cells (hMCs) using miR-339-5p mimics. (**A**) Reduced expression of *NEAT1_1/2* after OE_miR339-5p_ was detected after 24 or 48 h, shown as x-fold changes + SD for *NEAT1_1* (black bars + SD) and *NEAT1_2* (grey bars + SD), normalized to the expression after treatment with negative control RNA (NC_miR339-5p_); *n* = 4. (**B**) Cell migration was analyzed by wound healing assay by measuring the gap width, directly after removing the cell culture insert (0 h) and 4 h, 8 h, 12 h, and 24 h later. Black circles ± SD or grey triangles ± SD represent hMCs subjected to NC_miR339-5p_ and OE_miR339-5p_, respectively; *n* = 4. (**C**) Proliferation rate of hMCs after OE_miR339-5p_ (grey bar + SD) compared to NC_miR339-5p_ treated cells (black bar + SD). Proliferation was ascertained by a BrdU assay; *n* = 4. (**D**) Cell size in µm^2^ of hMCs after OE_miR339-5p_ (grey bar + SD) compared to NC_miR339-5p_ treated cells (black bar+ SD). Cell size measurement based on WGA immunostaining followed by morphometric analyses; *n* = 3. (**E**) x-fold change of mRNA expression of selecteup- or downregulated differentially expressed coding genes with predicted miR-339-5p binding site after KD*_NEAT1_* (black bars + SD) and OE_miR339-5p_ (grey bars + SD), respectively, compared to the corresponding NC*_NEAT1_* or NC_miR339-5p_, *n* = 3–4. (**F**) x-fold change of mRNA expression of selecteup- or downregulated differentially expressed coding genes without predicted miR-339-5p binding site after KD*_NEAT1_* (black bars + SD) and OE_miR339-5p_ (grey bars + SD) compared to the corresponding NC*_NEAT1_* or NC_miR339-5p_, *n* = 3–4. Significance was analyzed by Student’s *t*-tests. * *p* < 0.05; ** *p* < 0.01; *** *p* < 0.001; n.s. = not significant.

To analyze the role of miR-339-5p for mesangial physiology, the miRNA was overexpressed by using miR-339-5p mimics (OE_miR339-5p_; OE: overexpression). In contrast to KD*_NEAT1_* (Figure 2A), there was apparently no considerable effect on *NEAT1* expression after 24 h of miR-339-5p mimic application. After 48 h, however, both transcript variants exhibited a significant reduced expression (Figure 6A). To our surprise, this treatment resulted in a stronger diminution of *NEAT1* than KD*_NEAT1_* itself, especially for *NEAT1_1*. Next, we examined the relevance of miR-339-5p on hMC’s physiology by examining migration, proliferation, and cell size. Albeit less pronounced, OE_miR339-5p_ also lead to significantly enhanced migratory capabilities compared to NC cells (NC_miR339-5p_; Figure 6B and Figure S3). The gap was not entirely closed after 24 h. As with the KD*_NEAT1_*, the proliferation rate was significantly decreased, by 20% (Figure 6C). However, we could not ascertain an effect on cell size (Figure 6D). 

Since KD*_NEAT1_* as well as OE_miR339-5p_ lead to a reduced *NEAT1* expression (Figure 2A and Figure 6A), we wondered if both also had the same effect on the expression of other genes. The mRNA expression of eleven randomly selected genes after KD*_NEAT1_* and OE_miR339-5p_, respectively, was examined. After KD*_NEAT1_*, *CDKN1A*, *TGFB receptor 3* (*TGFBR3*), and pyruvate dehydrogenase kinase isoform 2 (*PDK2*) were upregulated while *CDK6* and insulin growth factor binding protein 3 (*IGFBP3*) were downregulated. These genes also carry a predicted miR-339-5p binding site (Table 1). All of them— except for *CDK6*, which showed no change—exhibited a significantly decreased expression after OE_miR339-5p_ (Figure 6E). This supports the reliability of our in-silico results, which suggest an potential targeting site for miR-339-5p.

*GNG4* and the transcription factor *E2F1*, which were increased expressed after KD*_NEAT1_*, as well as *TGFβ2*, *Gremlin1*, *CCND1*, and *CTGF*, which were less expressed after KD*_NEAT1_*, do not have a potential binding site for miR-339-5p. Interestingly, the expression of all was also significantly altered after OE_miR339-5p_ (Figure 6F). Yet, only *TGFβ2* and *CCND1* were influenced by KD*_NEAT1_* or OE_miR339-5p_, respectively, in the same manner. 

In summary, OE_miR339-5p_ leads to stronger migration but diminished proliferation in hMCs as well as to significant changes in gene expression, including *NEAT1*. A luciferase activity prompted an interaction of *NEAT1* and miR-339-5p, ensuing a reduced *NEAT1* expression. *NEAT1* was also decreased in various in-vitro diabetes experiments treating hMCs with TGFβ, TNAα, TG, or TM. Analyzing the effect of a KD*_NEAT1_*, we found many differentially expressed mRNAs and miRNAs. One of the latter was miR-339-5p, which was increased expressed. Like the OE_miR339-5p_, KD*_NEAT1_* resulted in more migration but less proliferation in hMCs. In-silico analyses support our findings. Our goal was to investigate the role of *NEAT1* in hMCs in various in-vitro diabetes models [34]. We identified the miRNA miR-339-5p as potential interaction partner for *NEAT1*. Based on our in-silico analyses and in-vitro experiments, we found various DECGs, which might be controlled by *NEAT1*, miR-339-5p, or both. Moreover, these DECGs play highly relevant roles in MCs during development and progression of DKD. Therefore, our results provide further insights in the role of *NEAT1* in DKD.

## 3. Discussion

*NEAT1* is involved in various mechanisms. It can attach to active chromatin sites depending on the transcriptional status of the corresponding gene [36]. As a structural component of nuclear paraspeckles [27], it binds RNAs causing their nuclear retention, which prevents their export to the cytoplasm and thus their translation [37]. *NEAT1* modulates gene expression by sponging numerous miRNAs [28,29,30,31,38]. The exact mechanism needs to be elucidated since *NEAT1* localizes to the nucleus while miRNAs have their place of action in the cytoplasm. However, two recently discovered findings could provide an explanation. In LPS-nigericin-stimulated murine immortalized bone marrow-derived macrophages, up to 15–20% of *Neat1* transcripts are translocated to the cytoplasm enhancing inflammasome activation [39]. Second, Castanotto et al. identified a stress-induced response complex called SIRC, which allows the relocation of miRNAs to the nucleus. Here, they revealed that miR-9 is transported back to the nucleus and then directly targets the lncRNA metastasis associated lung adenocarcinoma transcript 1 (*MALAT1*). Like *NEAT1*, *MALAT1* localizes in the nucleus allowing the formation of subcellular structures [40]. Others reported an additional mechanism for how *NEAT1* affects the level of cellular miRNAs: the 3′-end of *NEAT1_2* harbors a binding site for Microprocessor which facilitates miRNA processing [41]. 

In terms of physiology and pathology, various research groups determined *NEAT1* as a prognosis marker for poor survival rates and promoter of tumorigenesis [28,29,30]. With regard to DKD, others showed a positive and negative correlation, respectively, between elevated urinary *NEAT1* and various podocyte damage markers like synaptopodin and podocalyxin or GFR [42]. HG-exposed proximal tubular cells (HK-2) or murine MCs (mMCs) exhibited an increase in *NEAT1* expression in a dose- or time- dependent manner [31,43]. Contrarily to others, our data indicate that *NEAT1* expression is not affected by HG using an osmotic control and a species model, in which *NEAT1* is annotated (human or murine) [31,43,44].

Although hyperglycemic states are usually investigated to study DKD, one in-vitro model alone cannot reflect its complexity [34]. All further in-vitro DN models, tested in our approach, consistently point to a reduced *NEAT1* expression. Regarding TGFβ1, however, others showed that stimulation of various cell types with this cytokine resulted in an enhanced *NEAT1* expression [38,45]. Yet, this result can be appropriately put in the general context, with respect to the miscellaneous character of TGFβ. Although TGFβ has a cytostatic effect in many cell types, other cells like kidney fibroblasts and SMCs react with proliferation [46] while in hMCs, an apoptotic effect was demonstrated [47,48]. This is in line with the contradictory responses of MC to diabetic stimuli [15].

Circulating TNF receptors and TNFα in urine and serum are elevated in DCD and concomitant with protein excretion [49,50]. Although TNFα represents a key inducer of cell death, it strongly depends on the signaling molecules involved, as to which path will be chosen. Hereby, nuclear factor κB (NF-κB) and mitogen-activated protein kinase (MAPK) play a crucial role [51]. Related to MCs, TNFα was reported to serve as a relevant factor for apoptosis [52], but also for enhanced cell migration [53]. Our data revealed that TNFα stimulation leads to significantly decreased *NEAT1* expression and *NEAT1* silencing, and in turn, to less proliferation but more migration. NF-κB might be a direct link between TNFα and *NEAT1* since Zhou et al. demonstrated regulation of *NEAT1* expression via the NF-κB signaling pathway [54]. 

To our knowledge, there were no examinations regarding the effect of tunicamycin (TM) and thapsigargin (TG) on *NEAT1* expression so far. Treatment of hMCs with TG or TM for 24 h leads to a significant diminution of *NEAT1* expression. Others assessed the correlation between ER stress or cell death and the relative quantity of *NEAT1* RNA in gastric cancer tissues. While *NEAT1* expression was reduced, levels of ER stress and apoptosis marker proteins were elevated [55]. Taken together, the striking finding here and elsewhere is the described effects of *NEAT1* silencing or TGFβ1, TNFα, TG, or TM stimulation on migration, proliferation, and cell size (and *NEAT1* expression for the later), respectively, supporting the role of *NEAT1* as relevant factor for various regulatory mechanisms. However, the question of whether *NEAT1* has a positive or negative effect, seems to depend on the cell type and further mechanisms, which need to be elucidated in future investigations.

We conducted two microarrays after *NEAT1* silencing. The fact that two thirds of mRNAs were downregulated and two thirds of miRNAs were upregulated underlines the role of *NEAT1* as ceRNA [28,29,30,31]. However, others, who used a cancer cell line, observed that 69% of miRNAs showed decreased expression after KD*_NEAT1_*, assigning an implication in miRNA processing [41]. Here again, we may suppose that *NEAT1* functions variably depend on cell type or cell line. In our HMC experiments, exposure to various stimuli caused a reduced cellular *NEAT1* level—in contrast to others, who reported an increase in *NEAT1* expression after treatment with e.g., high glucose [43,44]. We also showed that HMCs exhibited less proliferation and cell size after KD*_NEAT1_*. This is in accordance with the RNA expression analysis, e.g., the increased *CDKN1A* or decreased *Ctgf* RNA expression, which is associated with apoptosis [56] and less ECM accumulation [57], respectively. This supports the conclusion that our HMC cell line responds to a diabetes-related stimulus rather with apoptosis and mesangiolysis than with proliferation and ECM production. Accordingly, *NEAT1* seems to be a relevant factor in determining the cellular response.

We also aimed to identify a miRNA, which might regulate this lncRNA, and thus affect mesangial physiology. Our data unveiled that miR-339-5p could be a notable candidate as our experiments and in-silico analyses suggest. So far, most experiments concerning miR-339-5p were conducted with cancer cells or tumor tissue, e.g., two studies focusing on p53-binding [58] protein mouse double minute 2 (MDM2), which is a direct target of miR-339-5p. Reduced Mdm2 expression results in increased amounts of p53 and p21 protein [59] as well as *CDKN1A* RNA [56] mediating apoptosis. Others identified miR-339-5p as biomarker for the outcome of a breast cancer therapy [60].

In a porcine epithelial cell line, however, miR-339-5p attenuated lipopolysaccharide induced p53 expression [61]. Afgar et al. directly compared the effect of OE_miR339_ on the methylation level of various tumor suppressor genes in a non-cancer cell line and two neoplastic cell lines. Only the latter exhibited a decreased methylation level after miR-339 treatment resulting in changes in cell cycle [62]. 

To summarize these aspects, the role of both *NEAT1* and miR-339-5p (often) depends on whether a cell (line) is neoplastic or non-neoplastic. Like with other cancers [28,29], *NEAT1* represents a sinister predictor for a poor outcome in osteosarcoma [30]. Thereby, it also serves as ceRNA for several miRNAs, including miR-339-5p [30]. This interaction promotes proliferation, migration, and invasion while inhibiting cell apoptosis [30,63]. In contrast to this, *NEAT1*, sponging miR-339-5p in mouse model of hypoxic-ischemic brain damage, resulted in relieving neuronal damage by elevating Homeobox A1 (HOXA1) expression [64].

Taken together, our data demonstrate the relevance of *NEAT1* and miR-339-5p for mesangial physiology. Both influence proliferation and migration as well as gene expression. In-silico investigations regarding GAD diseases, different terms of GO, and KEGG pathways substantiate this finding with respect to both KD*_NEAT1_* and OE_miR339-5p_. Therefore, identified terms such as type 2 diabetes, various GO BPs relating to proliferation as well as the GO terms extracellular matrix and cortical cytoskeleton, or p53 and TGFβ signaling pathway point to the significance of both ncRNAs in diabetes and MCs’ physiology. Additional qPCR analyses after OE_miR339-5p_ supported preceding in-silico studies further.

The two main limitations of this study are that only in-vitro investigations and in-silico analyses on predicted miRNA target sites of DECGs were conducted. Both are artificial and cannot cover the high complexity in vivo, but they also represent relevant tools, which allow the analysis of isolated aspects in more detail and with higher reproducibility. Human and animal studies can help to understand mechanisms in vivo. Nevertheless. we are aware that results and conditions in vivo might be different. While *NEAT1* is well conserved in humans and mice, other lncRNAs exhibit only a poor conservation. However, of importance in this context is the fact that various cell populations within the kidney but also from other tissues, interact with each other either directly via cell-cell contact or messenger molecules and vesicles. This is in particular the case for widely used hMCs, which are in continuous crosstalk with endothelial cells, podocytes, and immigrated immune cells. In-vitro analyses cannot reflect this entirely. 

Regarding the in-silico studies, genes with a predicted miR-339-5p binding site might not be targeted in vivo while other genes, which were negative for this, actually have a miR-339-5p targeting site. Furthermore, we could not examine the entire interaction network of *NEAT1*, miR-339-5p, and all coding genes. Nevertheless, in-silico analyses represent a powerful tool to correlate the data shown here and the large amount of already published data [65]. For our work, assumptions of cause and effect remain elusive. For example, both *CDKN1A*, with a predicted miR-339-5p binding site, and *E2F1*, without a predicted miR-339-5p binding site, showed enhanced expression after KD*_NEAT1_* but lower expression after OE_miR339-5p_ (Figure 6E,F). However, *E2F1* tightly controls the expression of *CDKN1A* [66]. Thus, the question arises whether the reduced *CDKN1A* expression is caused by interaction with miR-339-5p, by the diminished *E2F1* expression, or by a third reason. Nevertheless, our data suggest that the interplay of *NEAT1* and miR-339-5p influences mesangial gene expression and thus kidney physiology.

## 4. Materials and Methods

### 4.1. General Information

All experiments, if not stated otherwise, were conducted according to manufacturer’s instructions. Chemicals, kits, as well as software and devices, together with their manufacturers as well as internet addresses of databases and online tools are shown in Supplementary Tables S16–S18.

### 4.2. Cell Culture Maintenance

In-vitro experiments were conducted with an established immortalized hMC line. This cell line was purchased from Clonetics Corp. (San Diego, CA, USA) and then maintained and characterized as described previously [67]. Cells were cultured under sterile conditions at 37 °C in an atmosphere of 5% CO_2_/95% air, using hMC medium consisting of Dulbecco’s modified Eagle’s medium (DMEM 21855) supplemented with 100 U/mL penicillin, 100 µg/mL streptomycin (P/S), and 10% fetal calf serum (FCS).

### 4.3. ISH Localization in hMCs

In a 24 well plate, hMCs were allowed to adhere to poly-L-lysine coated cover slips overnight, prior to fixation with 4% formaldehyde for 30 min. To label and localize *NEAT1* RNA, we used the ViewRNA™ ISH Cell Assay Kit including the corresponding probes set for human *NEAT1* and an AxioStar Plus Observer Z1 fluorescence microscope. Target RNA was visualized with fluorescent label probes at excitation wavelengths of 488 nm. Nuclei were stained with 4′,6-diamidino-2-phenylindole (DAPI).

### 4.4. In-Vitro Stimulation Experiments

HMCs were seeded at 150,000 cells per well in 6-well plates and starved for 72 h by reducing the FCS content to 0.1% (hMC medium_0.1%_) before performing stimulation experiments (retaining cells in hMC medium_0.1%_). Cells were exposed to 30 mM glucose or mannitol (24.5 mM + 5.5 mM glucose in hMC medium_0.1%_) as osmotic control for 0.5 to 24 h. Recombinant human TGFβ1 and TNFα were used at final concentrations of 100 or 10 ng/mL, respectively, while hMC medium_0.1%_ alone served as reference. For cells treated with 100 ng/mL TG or 100 nM TM, the corresponding amount of dimethyl sulfoxide (DMSO) was added to the control approach. All experiments were repeated at least three times.

### 4.5. NEAT1 Knockdown

In a double transfection procedure, 1.5 × 10^5^ hMCs were reverse transfected in solution and allowed to adhere for 48 h in a 6-well plate. Then, a second transfection mixture was added and cell were cultured for another 24 or 48 h. To generate the transfection complex, 4 µL *NEAT1* or NC siPOOLs were pre-mixed in 489 μL Opti-MEM with 7 μL Lipofectamin RNAiMAX to reach a final concentration of 6.67 nM. After an incubation time of 15 min, 2.5 mL of FCS- and P/S-free medium was added to the transfection mixture.

### 4.6. Overexpression of miR-339-5p

In a reverse transfection procedure, 1.5 × 10^5^ hMCs were added per well (6-well plate) containing a mixture of 4 µL Lipofectamin, 4.8 µL NC or miR339-5p mimic and 491.2 µL Opti-MEM. Cells were incubated for 48 h with a final amount of 19.2 nM NC or mimic before harvesting for RNA expression analysis.

### 4.7. Proliferation Assay

*NEAT1* was knocked down as described above. Then, 24 h after the second KD, hMCs were detached using accutase, and transferred in 96-well plates with a density of 5000 cells per well. At 24 h after cell seeding, a BrdU Cell Proliferation Assay was performed. MiR-339-5p mimic treatment was conducted, as described above, but for 30 h. Subsequently, hMCs were harvested, seeded at 4000 cells per well in 96-well plates, and allowed to adhere overnight before applying the BrdU Cell Proliferation Assay Kit. Proliferation assay was performed in triplicate and repeated four or five times.

### 4.8. Wound Healing Assay

To analyze hMCs’ migration capabilities after KD*_NEAT1_* or OE_miR339-5p_, one 2-well silicone cell culture insert was placed on the bottom of each well in a 12-well plate, before seeding 8.0 × 10^4^ and 1.5 × 10^5^ cells, respectively, in a reverse transfection procedure. At 24 h after *NEAT1* silencing, a second KD of the lncRNA was conducted, as described above. Then, 24 h after the second KD*_NEAT1_* or 48 h after miR339-5p mimic application, the cell culture inserts were removed, leaving a gap of 800 µm. The full width of the gap was documented immediately using an AxioStar Plus Observer Z1 microscope. Further records were taken at the same positions 4, 8, 12, and 24 h later. The assay was repeated three or four times.

### 4.9. Cell Size Measurement

KD*_NEAT1_* and OE_miR339-5p_ were carried out as described above. Subsequently, cells were fixed with 4% formaldehyde and incubated with the Alexa Fluor 594 conjugated anti-wheat germ agglutinin (WGA) antibody to stain the cell membrane. Nuclei were visualized with DAPI. This experiment was repeated three or four times, analyzing 60–70 randomly selected cells in a blinded manner. Based on WGA membrane staining, cell size was measured using HistoQuest software.

### 4.10. Cloning of Luciferase Reporter Gene Vectors and Luciferase Assay

We used PmeI restriction endonuclease linearized pmirGLO luciferase vector as starting point to generate plasmids containing *NEAT1* WT or MUT target sites (sequences: Supplementary Table S19). These sites were cloned using the NEBuilder HiFi DNA Assembly kit, conducting the manufacturer‘s protocol “Bridging dsDNA with a ssDNA Oligo”. Single stranded DNA oligos contained miR-339-5p WT or MUT target sequences flanked by 25–30 bases homologous to the vector sequence.

We seeded 1.2 × 10^5^ hMCs in 950 µL hMC medium per well of a 12-well plate. One transfection mixture consisted of 2 µL Lipofectamin, 2.4 µL NC or miR339-5p mimic, and 45.6 µL Opti-MEM, which was added to the cells for 8 h and then replaced by fresh hMC medium. The second transfection mixture, composed of 1.5 µg corresponding luciferase reporter gene vector and 5 µL Attractene Transfection Reagent in Opti-MEM ad 125 µL, was added for 6 h and then replaced by fresh hMC medium for another 12 h, before conducting a Dual-Glo^®^ Luciferase Assay. Luciferase and renilla activities were measured by Tecan infinite 200 pro reader.

### 4.11. Expression Analysis of DECGs and DEmiRNAs

To extract total RNA and miRNA, respectively, hMCs were lysed by adding the corresponding buffer of the NucleoSpin RNA Plus Kit or the miRNeasy Mini Kit. Each RNA isolation procedure always included an on-column DNase I treatment. RNA concentration was determined by means of the NanoDrop 2000c spectrophotometer. For cDNA synthesis, we used a M-MLV reverse transcriptase system including random primers and RNasin for total RNA or the miRCURY LNA RT kit for miRNAs.

To analyze and validate gene expression, qPCR experiments were performed applying the QuantiTect SYBR Green PCR kit or miRCURY LNA SYBR Green PCR kit. All samples were run in triplicate using the ViiA 7 Real-Time PCR System. The xFC was calculated based on the 2^−ΔΔCT^ method with peptidylprolyl isomerase B or hsa-5S-rRNA as reference genes. Primers for mRNA/lncRNA genes (sequences: Supplementary Table S20) were designed using Primer3 software. We only used oligonucleotides with an efficiency between 90 and 110%. Validation of miRNA expression was conducted with miCURY LNA miRNA PCR assays.

### 4.12. Clariom™ S Assay Human and GeneChip™ miRNA 4.0 Array

Changes in mRNA or miRNA expression were examined my means of the Clariom™ S Assay human and GeneChip™ miRNA 4.0 Array, respectively. The technical implementation was conducted at the Center of Excellence for Fluorescent Bioanalytics” (Regensburg, Germany; www.kfb-regensburg.de (accessed on 24 July 2018)) as described previously [22]. The data were obtained as CEL files and imported into the Transcriptome Analysis Console (TAC) software 4.0 to summarize probe set signals based on the SST-RMA algorithm. Coding genes with a mean channel intensity < 100 were excluded (which corresponds to a log 2 transformed intensity of 7.64). Further filter criteria were a xFC > 2 or <−2 and a false discovery rate (FDR) adjusted *p* value < 0.05. Only miRNAs with a mean channel intensity > 10 were considered (log 2 value = 3.32). Cut-offs for differential miRNA expression were defined as xFC > 1.5 or <−1.5 and *p* value < 0.05. Statistical analysis was based on the Fisher-exact test. Proceeding from the data obtained, the TAC software tool, miR-Interactions“ was used to identify mRNA-miRNA interaction networks.

### 4.13. Functional Enrichment Analyses

In terms of GAD Disease Analysis, GO categories, and KEGG pathways, the Database for Annotation, Visualization, and Integrated Discovery (DAVID) v6.8 was used to identify gene clustering and enriched pathways. An expression analysis systematic explorer (EASE) score and a *p* value < 0.05 were considered as significant. The online tool DIANA miRPath v.3.0 [68] was applied to conduct miRNA functional analyses using KEGG or Gene Ontology annotation, with a significance level of <0.05.

### 4.14. Statistics

Statistical analysis of microarray data was automatically conducted by TAC software 4.0. The database DAVID also provided statistical exploration for functional enrichment analyses. The remaining statistical analyses were performed using SPSS Statistics Version 21. Normal distribution or homogeneity of variances were ascertained by Shapiro–Wilk and Levene’s test, respectively. For two independent means, Student’s *t*-test was carried out. To measure the overall significance of differences in more than two means, an analysis of variance (ANOVA) was conducted, before using Student’s *t*-tests for post hoc pairwise comparisons. To determine if any term of GAD Disease, GO category, or KEGG pathways was statistically significant over-represented, we conducted hypergeometric testing. Statistical difference was set at the 5% level of probability.

## Figures and Tables

**Table 1:** x-fold changes (xFC) of DECGs with predicted miR339-5p binding site.

Upregulated DECGs		Downregulated DECGs	
Name	xFC	Name	xFC
SERPINE1	−11.82	EPB41	2.03
EDN1	−11.13	CBFA2T2	2.07
RGS4	−4.77	RAB15	2.08
IGFBP3	−4.19	PDK2	2.17
SKP2	−3.55	CUX1	2.39
NT5DC3	−3.3	MXD4	2.54
DDAH1	−3.17	ITPK1	2.55
VGLL3	−3.09	SMC1A	2.65
FLNA	−3.01	CDKN1A	2.75
ENTPD7	−2.97	TGFBR3	2.89
LBH	−2.95	BTBD2	2.92
DCP2	−2.93	MAN2A2	3.19
SRGN	−2.91	OAS3	7.21
HMGCR	−2.66		
CDK6	−2.57		
NAV3	−2.53		
LBR	−2.45		
GSKIP	−2.4		
RUFY2	−2.29		
ATXN1	−2.2		
TACC1	−2.2		
PDE4D	−2.19		
COPZ1	−2.19		
CCDC50	−2.17		
DUSP5	−2.16		
TMEM2	−2.08		
ENC1	−2.03		

## Data Availability

The datasets used and/or analyzed during the current study are available from the corresponding author on reasonable request.

## Supplementary Materials

The following supporting information can be downloaded at: https://www.mdpi.com/article/10.3390/ncrna8040052/s1, Table S1: Differentially expressed coding genes after 24 h of silencing *NEAT1*, Table S2: Differentially expressed coding genes after 48 h of silencing *NEAT1*, Table S3: Significantly enriched GAD diseases based on all DECGs, Table S4: Significantly enriched GO BP (direct) based on all DECGs, Table S5: Significantly enriched GO CC (direct) based on all DECGs, Table S6: Significantly enriched GO MF (direct) based on all DECGs, Table S7: Significantly enriched KEGG pathways based on all DECGs, Table S8: Differentially expressed miRNAs after 24 h of silencing *NEAT1*, Table S9: Differentially expressed miRNAs after 48 h of silencing *NEAT1*, Table S10: Significantly enriched GO BP based on DEmiRNAs, Table S11: Significantly enriched KEGG pathways based on DEmiRNAs, Table S12: Identification of *NEAT1* binding miRNAs using the three databases: RNA22, mirDIP, and Starbase, Table S13: Significantly enriched GAD diseases based on DECGs with potential miR-339-5p targeting site, Table S14: Significantly enriched GO BP based on DECGs with potential miR-339-5p targeting site, Table S15: Significantly enriched GO CC based on DECGs with potential miR-339-5p targeting site, Table S16: Chemicals, agents, kits, as well as software and devices with their manufacturers, Table S17: Software and devices, Table S18: Data bases and online tools, Table S19: Single stranded DNA oligo sequences for cloning of luciferase reporter gene vectors, Table S20: Primer sequences, Figure S1: x-fold changes of *NEAT1_1* and *NEAT1_2* expression after stimulation. Figure S2: Migration assay after KD*_NEAT1_* in HMCs. Figure S3: Migration assay after OE_miR339-5p_ in HMCs.
