# Supplementary material for: The Interplay of NEAT1 and miR-339-5p Influences on Mesangial Gene Expression and Function in Various Diabetic-Associated Injury Models
Source: Noncoding RNA. 2022 Jul 13;8(4):52. doi: 10.3390/ncrna8040052 (PMC9326603; doi:10.3390/ncrna8040052)
Supplement: Supplementary file 1 [file ncrna-08-00052-s001.zip › ncrna-1767926-supplementary.pdf]

Supplementary Table S1:

Differentially expressed coding genes after 24 h of silencing NEAT1

**Gene**	**x-fold change**	**FDR p value**	**Description**
EDN1	-11.13	0.0006	Endothelin 1
CTGF	-5.87	0.0001	Connective tissue growth factor
ACTC1	-5.81	0.0147	Actin, alpha, cardiac muscle 1
RGS4	-4.77	0.0149	Regulator of g-protein signaling 4
INHBA	-4.53	0.0001	Inhibin beta a
THBS1	-4.22	0.0415	Thrombospondin 1
ADIPOR1	-3.8	0.0055	Adiponectin receptor 1
GNPNAT1	-3.67	0.0077	Glucosamine-phosphate n-acetyltransferase 1
SKP2	-3.55	0.0006	S-phase kinase-associated protein 2, e3 ubiquitin protein ligase
TRPC4	-3.5	0.0212	Transient receptor potential cation channel, subfamily c, member 4
MYOCD	-3.29	0.0187	Myocardin
DDAH1	-3.17	0.0385	Dimethylarginine dimethylaminohydrolase 1
VGLL3	-3.09	0.0208	Vestigial-like family member 3
LPCAT2	-3.04	0.01	Lysophosphatidylcholine acyltransferase 2
GREM1	-3.04	0.0208	Gremlin 1, dan family bmp antagonist [source:hgnc symbol
SH3BGRL2	-3.02	0.0147	Sh3 domain binding glutamate-rich protein like 2
ENTPD7	-2.97	0.0208	Ectonucleoside triphosphate diphosphohydrolase 7
TGFB2	-2.97	0.0147	Transforming growth factor beta 2
LBH	-2.95	0.0087	Limb bud and heart development
VAT1L	-2.94	0.0208	Vesicle amine transport 1-like
ARID5B	-2.86	0.0147	At rich interactive domain 5b (mrf1-like)
SGK1	-2.86	0.0208	Serum/glucocorticoid regulated kinase 1
NECAP1	-2.78	0.01	Necap endocytosis associated 1
CCND1	-2.76	0.0258	Cyclin d1
GTF2E1	-2.7	0.009	General transcription factor iie subunit 1
PDE1C	-2.64	0.0147	Phosphodiesterase 1c, calmodulin-dependent 70kda
ADM	-2.61	0.0335	Adrenomedullin
ST6GALNAC5	-2.6	0.0188	St6 (alpha-n-acetyl-neuraminyl-2,3-beta-galactosyl-1,3)-n-acetylgalactosaminide alpha-2,6-sialyltransferase 5
CDK6	-2.57	0.009	Cyclin-dependent kinase 6
TIMP3	-2.54	0.0385	Timp metallopeptidase inhibitor 3
NAV3	-2.53	0.0208	Neuron navigator 3
CRIM1	-2.53	0.0198	Cysteine rich transmembrane bmp regulator 1 (chordin-like)
YTHDF3	-2.51	0.0255	Yth n(6)-methyladenosine rna binding protein 3

ZNF460	-2.5	0.0149	Zinc finger protein 460
ADAM19	-2.49	0.0147	Adam metallopeptidase domain 19
LBR	-2.45	0.0208	Lamin b receptor
TROVE2	-2.45	0.0208	Trove domain family, member 2
GSTCD	-2.42	0.0055	Glutathione s-transferase, c-terminal domain containing
GSKIP	-2.40	0.0208	Gsk3b interacting protein
AMIGO2	-2.40	0.0314	Adhesion molecule with ig-like domain 2
PAQR3	-2.35	0.0090	Progestin and adipoq receptor family member iii
FAM3C	-2.34	0.0259	Family with sequence similarity 3, member c
ERRFI1	-2.34	0.0210	Erbb receptor feedback inhibitor 1
STARD7	-2.33	0.0323	Star-related lipid transfer domain containing 7
GOLT1B	-2.31	0.0121	Golgi transport 1b
UBASH3B	-2.25	0.0424	Ubiquitin associated and sh3 domain containing b
HN1	-2.24	0.0208	Hematological and neurological expressed 1
IER3	-2.23	0.0209	Immediate early response 3
ATXN1	-2.20	0.0448	Ataxin 1
TACC1	-2.20	0.0323	Transforming, acidic coiled-coil containing protein 1
DUSP5	-2.16	0.0208	Dual specificity phosphatase 5
SLC20A2	-2.12	0.0147	Solute carrier family 20 (phosphate transporter), member 2
CYR61	-2.09	0.0284	Cysteine-rich, angiogenic inducer, 61
TMEM2	-2.08	0.0208	Transmembrane protein 2
ENC1	-2.03	0.0288	Ectodermal-neural cortex 1 (with btb domain)
AP2A1	2.01	0.0147	Adaptor-related protein complex 2, alpha 1 subunit
PBK	2.04	0.0149	Pdz binding kinase
GATS	2.05	0.0475	Gats, stromal antigen 3 opposite strand
CBFA2T2	2.07	0.0385	Core-binding factor, runt domain, alpha subunit 2
TSPAN3	2.07	0.0252	Tetraspanin 3
MLST8	2.10	0.0208	Mtor associated protein, lst8 homolog
PDGFA	2.17	0.0055	Platelet-derived growth factor alpha polypeptide
PDK2	2.17	0.0208	Pyruvate dehydrogenase kinase, isozyme 2
COA3	2.26	0.0149	Cytochrome c oxidase assembly factor 3
C3orf14	2.34	0.0275	Chromosome 3 open reading frame 14
CUX1	2.39	0.0243	Cut-like homeobox 1
WBP1L	2.41	0.0263	Ww domain binding protein 1-like
SLC25A10	2.42	0.0208	Solute carrier family 25 (mitochondrial carrier dicarboxylate transporter), member 10
CTSH	2.44	0.0399	Cathepsin h
RBM8A	2.47	0.0208	Rna binding motif protein 8a
JUND	2.49	0.0285	Jun d proto-oncogene
MXD4	2.54	0.0147	Max dimerization protein 4
ITPK1	2.55	0.0335	Inositol-tetrakisphosphate 1-kinase
DHRS11	2.59	0.0077	Dehydrogenase/reductase (sdr family) member 11

ARL6IP1	2.62	0.0208	Adp-ribosylation factor like gtpase 6 interacting protein 1
RBPMS2	2.67	0.0208	Rna binding protein with multiple splicing 2
CDKN1A	2.75	0.0208	Cyclin-dependent kinase inhibitor 1a (p21, cip1)
NDUFB1	2.89	0.0390	Nadh dehydrogenase (ubiquinone) 1 beta subcomplex, 1, 7kda
LRRC8C	2.92	0.0147	Leucine rich repeat containing 8 family, member c
BTBD2	2.92	0.0149	Btb (poz) domain containing 2
H1F0	3.03	0.0316	H1 histone family, member 0
NARF	3.52	0.0255	Nuclear prelamin a recognition factor
C1QL1	4.00	0.0400	Complement component 1, q subcomponent-like 1
KLF2	4.09	0,0353	Kruppel-like factor 2

Supplementary Table S2:

Differentially expressed coding genes after 48 h of silencing NEAT1

**Gene**	**x-fold change**	**FDR p value**	**Description**
ACTG2	-24.46	0.0065	Actin, gamma 2, smooth muscle, enteric
TAGLN	-19.67	0.0083	Transgelin
CALD1	-11.89	0.0186	Caldesmon 1
SERPINE1	-11.82	0.0406	Serpin peptidase inhibitor, clade e (nexin, plasminogen activator inhibitor type 1), member 1
RGS4	-9.22	0.0114	Regulator of g-protein signaling 4
GLIPR1	-7.14	0.0454	Gli pathogenesis-related 1
THBS1	-6.54	0.0111	Thrombospondin 1
VAT1L	-6.21	0.0034	Vesicle amine transport 1-like
TPM1	-5.57	0.0113	Tropomyosin 1 (alpha)
ADM	-5.03	0.0428	Adrenomedullin
CCND1	-4.76	0.0186	Cyclin d1
GNPNAT1	-4.49	0.0118	Glucosamine-phosphate n-acetyltransferase 1
ENTPD7	-4.36	0.0146	Ectonucleoside triphosphate diphosphohydrolase 7
IGFBP3	-4.19	0.0321	Insulin like growth factor binding protein 3
TGM2	-4.10	0.0262	Transglutaminase 2
GREM1	-4.02	0.013	Gremlin 1, dan family bmp antagonist [source:hgnc symbol
PDE1C	-3.92	0.0031	Phosphodiesterase 1c, calmodulin-dependent 70kda
CYR61	-3.83	0.0113	Cysteine-rich, angiogenic inducer, 61
CTGF	-3.57	0.0034	Connective tissue growth factor
ACLY	-3.35	0.0215	Atp citrate lyase
TGFB2	-3.33	0.0138	Transforming growth factor beta 2
NT5DC3	-3.30	0.0118	5-nucleotidase domain containing 3
ENC1	-3.30	0.0142	Ectodermal-neural cortex 1 (with btb domain)
ZNF460	-3.23	0.0102	Zinc finger protein 460
MEST	-3.14	0.0113	Mesoderm specific transcript
FRMD6	-3.12	0.0111	Ferm domain containing 6
RHOJ	-3.04	0.0083	Ras homolog family member j
ANTXR2	-3.04	0.0296	Anthrax toxin receptor 2
HMGCS1	-3.02	0.0376	3-hydroxy-3-methylglutaryl-coa synthase 1 (soluble)
SBNO1	-3.01	0.0064	Mir8072
FLNA	-3.01	0.0354	Filamin a, alpha
ADIPOR1	-2.97	0.0113	Adiponectin receptor 1
DCP2	-2.93	0.0239	Decapping mrna 2
SRGN	-2.91	0.0198	Serglycin
CRIM1	-2.84	0.0169	Cysteine rich transmembrane bmp regulator 1 (chordin-like)
NECAP1	-2.81	0.0055	Necap endocytosis associated 1

MET	-2.80	0.0179	Met proto-oncogene, receptor tyrosine kinase
SKP2	-2.79	0.0034	S-phase kinase-associated protein 2, e3 ubiquitin protein ligase
PPP2CB	-2.74	0.0146	Protein phosphatase 2, catalytic subunit, beta isozyme
SEMA3C	-2.74	0.0118	Sema domain, immunoglobulin domain (ig), short basic domain, secreted, (semaphorin) 3c
CLDN11	-2.69	0.0064	Claudin 11
UBASH3B	-2.68	0.0468	Ubiquitin associated and sh3 domain containing b
HN1	-2.67	0.0064	Hematological and neurological expressed 1
HMGCR	-2.66	0.0156	3-hydroxy-3-methylglutaryl-coa reductase
FDFT1	-2.65	0.0113	Farnesyl-diphosphate farnesyltransferase 1
CDK6	-2.64	0.0064	Cyclin-dependent kinase 6
GOLT1B	-2.59	0.0064	Golgi transport 1b
LBR	-2.57	0.0064	Lamin b receptor
LIMS1	-2.51	0.0419	Lim and senescent cell antigen-like domains 1
UHMK1	-2.51	0.0031	U2af homology motif (uhm) kinase 1
PAQR3	-2.50	0.0064	Progestin and adipoq receptor family member iii
FHL2	-2.48	0.0158	Four and a half lim domains 2
AVEN	-2.45	0.0242	Apoptosis, caspase activation inhibitor
MSMO1	-2.45	0.0173	Methylsterol monooxygenase 1
SMS	-2.36	0.0064	Spermine synthase
RNF24	-2.35	0.0183	Ring finger protein 24
TROVE2	-2.35	0.0168	Trove domain family, member 2
LPCAT2	-2.32	0.0293	Lysophosphatidylcholine acyltransferase 2
SSR3	-2.31	0.0138	Signal sequence receptor, gamma (translocon-associated protein gamma)
RUFY2	-2.29	0.0102	Run and fyve domain containing 2
CAV1	-2.23	0.0168	Caveolin 1
PDE4D	-2.19	0.0076	Phosphodiesterase 4d, camp-specific
COPZ1	-2.19	0.0360	Coatomer protein complex subunit zeta 1
BMPR1A	-2.18	0.0064	Bone morphogenetic protein receptor type ia
CCDC50	-2.17	0.0434	Coiled-coil domain containing 50
SLC38A6	-2.16	0.0466	Solute carrier family 38, member 6
UBE4A	-2.15	0.0376	Ubiquitination factor e4a
STX3	-2.14	0.0064	Syntaxin 3
SPDL1	-2.12	0.0321	Spindle apparatus coiled-coil protein 1
SQLE	-2.11	0.0111	Squalene epoxidase
KIF5C	-2.11	0.0495	Kinesin family member 5c
ADSS	-2.08	0.0064	Adenylosuccinate synthase
ARHGEF28	-2.08	0.0171	Rho guanine nucleotide exchange factor 28
HIPK1	-2.08	0.0436	Homeodomain interacting protein kinase 1
WDR36	-2.07	0.0115	Wd repeat domain 36
SLC20A2	-2.06	0.0404	Solute carrier family 20 (phosphate transporter), member 2

SECISBP2L	-2.04	0.0405	Secis binding protein 2-like
GINS1	-2.03	0.0490	Gins complex subunit 1 (psf1 homolog)
GTF2E1	-2.00	0.0295	General transcription factor iie subunit 1
ARL5B	-2.00	0.0400	Adp-ribosylation factor like gtpase 5b
EPB41	2.03	0.0064	Erythrocyte membrane protein band 4.1
PSME1	2.05	0.0321	Proteasome activator subunit 1
UNK	2.08	0.0414	Unkempt family zinc finger
RAB15	2.08	0.0259	Rab15, member ras oncogene family
SDC3	2.1.	0.0241	Syndecan 3
TMEM106C	2.14	0.0434	Transmembrane protein 106c
GNA11	2.16	0.0329	Guanine nucleotide binding protein (g protein), alpha 11 (gq class)
PRDX2	2.16	0.0376	Peroxiredoxin 2
MOV10	2.17	0.0474	Mov10 risc complex rna helicase
ARL6IP1	2.18	0.0388	Adp-ribosylation factor like gtpase 6 interacting protein 1
CUX1	2.25	0.0113	Cut-like homeobox 1
C1orf21	2.26	0.0167	Chromosome 1 open reading frame 21
MLST8	2.28	0.0064	Mtor associated protein, lst8 homolog
DNPH1	2.33	0.0168	2-deoxynucleoside 5-phosphate n-hydrolase 1
C1QL4	2.39	0.0329	Complement component 1, q subcomponent-like 4
KCNIP3	2.45	0.0115	Kv channel interacting protein 3, calsenilin
HOMER2	2.46	0.0337	Homer scaffolding protein 2
AP2A1	2.51	0.0055	Adaptor-related protein complex 2, alpha 1 subunit
NUP210	2.58	0.0459	Nucleoporin 210kda
KIAA0101 CSNK1G1	2.58	0.0458	Casein kinase 1, gamma 1
SMC1A	2.65	0.0186	Structural maintenance of chromosomes 1a
COA3	2.65	0.0156	Cytochrome c oxidase assembly factor 3
GATS	2.68	0.0198	Gats, stromal antigen 3 opposite strand
MDK	2.68	0.0228	Midkine (neurite growth-promoting factor 2)
NCOA7	2.80	0.0329	Nuclear receptor coactivator 7
ITPK1	2.83	0.0146	Inositol-tetrakisphosphate 1-kinase
TGFBR3	2.89	0.0437	Transforming growth factor beta receptor iii
BTBD2	3.01	0.0113	Btb (poz) domain containing 2
E2F1	3.05	0.0034	E2f transcription factor 1
TEAD2	3.07	0.0227	Tea domain family member 2
SAMHD1	3.13	0.0374	Sam domain and hd domain 1
H1F0	3.14	0.0179	H1 histone family, member 0
MAN2A2	3.19	0.0214	Mannosidase, alpha, class 2a, member 2
PIK3R2 IFI30	3.24	0.0205	IPhosphoinositide-3-kinase, regulatory subunit 2 (beta) interferon, gamma-inducible protein 30
JUND	3.25	0.0295	Jun d proto-oncogene

SEMA6B	3.48	0.0146	Sema domain, transmembrane domain (tm), and cytoplasmic domain, (semaphorin) 6b
GP1BB SEPT5	3.81	0.0376	Glycoprotein Ib (platelet), beta polypeptide septin 5
GNG4	3.88	0.0341	Guanine nucleotide binding protein (g protein), gamma 4
LRRC8C	3.92	0.0064	Leucine rich repeat containing 8 family, member c
CDKN1A	4.21	0.0121	Cyclin-dependent kinase inhibitor 1a (p21, cip1)
PLSCR1	4.40	0.0468	Phospholipid scramblase 1
CPNE2	4.56	0.0415	Copine ii
WBP1L	4.62	0.0034	Ww domain binding protein 1-like
OAS3	7.21	0.0064	2-5-oligoadenylate synthetase 3
C1QL1	10.20	0.0169	Complement component 1, q subcomponent-like 1
MX1	21.65	0.0474	Mx dynamin-like gtpase 1

Supplementary Table S3:

Significantly enriched GAD diseases based on all DECGs

Term	P value	Count	Genes
Ovarian cancer	$3.25*10^{-9}$	17	DUSP5, CDKN1A, TGFB2, IGFBP3, MX1, SERPINE1, INHBA, CYR61, CTGF, H1F0, CDK6, CCND1, OAS3, E2F1, TIMP3, SKP2, MEST
Plasma HDL cholesterol (HDL-C) levels	0.002	9	SQLE, ACLY, EDN1, HMGCS1, PIK3R2, MSMO1, HMGCR, ADIPOR1, FDFT1
Type 2 Diabetes| edema	0.003	34	AVEN, CDKN1A, TAGLN, SERPINE1, ADM, HMGCR, ADIPOR1, THBS1, RGS4, GLIPR1, FLNA, TIMP3, CTSH, SKP2, LBR, TEAD2, FDFT1, DUSP5, TGFB2, MYOCD, EDN1, PDE4D, CAV1, IGFBP3, TPM1, TGFBR3, SQLE, ACTC1, CDK6, NAV3, DDAH1, RAB15, LPCAT2, SGK1
Lung cancer	0.003	13	CDKN1A, EDN1, CAV1, IGFBP3, MX1, SERPINE1, HMGCR, CTGF, CCND1, RAB15, CTSH, MEST, MET
Bone mineral density	0.005	11	TGFBR3, GREM1, CDKN1A, TGFB2, CDK6, CCND1, IGFBP3, E2F1, ADIPOR1, BMPR1A, CTGF
Atherosclerosis	0.006	10	EDN1, TGFB2, PDE4D, CAV1, IGFBP3, SERPINE1, PDGFA, HMGCR, ADIPOR1, THBS1
Colorectal cancer	0.006	11	GREM1, CDKN1A, CCND1, MDK, CAV1, IGFBP3, SERPINE1, HMGCR, INHBA, ADIPOR1, THBS1
Cholesterol, HDL	0.007	11	SBNO1, PDE4D, OAS3, TPM1, ITPK1, ARID5B, ADM, NCOA7, HMGCR, SGK1, CTGF
Bladder cancer	0.010	12	CDKN1A, EDN1, CDK6, CCND1, RAB15, CAV1, IGFBP3, MX1, CTSH, HMGCR, MEST, MET
Glaucoma, open-angle	0.011	4	CDKN1A, WDR36, CAV1, SERPINE1
Hepatopulmonary syndrome|liver cirrhosis	0.012	5	EDN1, CAV1, SERPINE1, THBS1, BMPR1A
Mouth neoplasms|precancerous conditions	0.012	3	CDKN1A, CDK6, CCND1
Chronic obstructive pulmonary disease	0.015	10	EDN1, CCND1, RAB15, CAV1, IGFBP3, MX1, CTSH, HMGCR, MEST, MET
Sleep	0.018	4	EPB41, PDE4D, ITPK1, NCOA7
Type 2 diabetes	0.019	9	GREM1, EDN1, DDAH1, IGFBP3, SERPINE1, HMGCR, ADIPOR1, KLF2, TGM2
Amyotrophic lateral sclerosis|anoxia|	0.023	3	CAV1, SERPINE1, INHBA
Glaucoma	0.024	3	CDKN1A, EDN1, WDR36

Hepatitis c, chronic|liver cirrhosis	0.026	4	TGFBR3, TGFB2, MX1, TIMP3
Heart failure	0.028	9	ADAM19, TGFBR3, EDN1, FRMD6, ATXN1, ARHGEF28, NCOA7, INHBA, SGK1
Bone density osteoporosis	0.028	2	TGFB2, SERPINE1
Obesity	0.030	7	TGFBR3, TGFB2, IGFBP3, SERPINE1, TACC1, INHBA, BMPR1A
Stroke	0.030	11	ATXN1, CUX1, YTHDF3, UBASH3B, TRPC4, PDE4D, SERPINE1, HMGCR, ADSS, VAT1L, C1QL1
Kidney dysfunction	0.037	2	EDN1, SERPINE1
Dupuytren's disease	0.037	2	TGFBR3, TGFB2
Squamous cell carcinoma of the head and neck	0.037	2	CDKN1A, CCND1
Retinopathy, diabetic	0.038	3	EDN1, SERPINE1, TIMP3
Oral cancer	0.049	3	CDKN1A, CCND1, SERPINE1

Enrichment analysis based on all coding genes expressed by hMCs (mean channel intensity > 100, according to Clariom™ S Assay); GAD: Genetic Association Database; EASE (expression analysis systematic explorer) score < 0.05; $p < 0.05$

Supplementary Table S4:

Significantly enriched GO BP (direct) based on all DECGs

Term	P value	Count	Genes
Negative regulation of apoptotic process	$2.57*10^{-12}$	14	AVEN, CDKN1A, ARL6IP1, AMIGO2, FHL2, THBS1, CYR61, GREM1, PRDX2, ACTC1, CTSH, FLNA, IER3, TGM2
Positive regulation of smooth muscle cell proliferation	$2.08*10^{-12}$	6	EDN1, RBPMS2, HMGCR, SKP2, THBS1, TGM2
Positive regulation of gene expression	$1.39*10^{-12}$	11	TGFB2, PLSCR1, ACTC1, CDK6, CAV1, E2F1, CTSH, INHBA, ACTG2, CTGF, LIMS1
Positive regulation of angiogenesis	$6.23*10^{-11}$	7	GREM1, DDAH1, SERPINE1, CTSH, ADM, HIPK1, THBS1
Platelet degranulation	$4.16*10^{-10}$	8	SRGN, TGFB2, SERPINE1, PDGFA, TIMP3, FLNA, FAM3C, THBS1
Cholesterol biosynthetic process	$1.08*10^{-10}$	7	SQLE, ACLY, HMGCS1, MSMO1, HMGCR, LBR, FDFT1
Cell cycle arrest	0.002	7	CDKN1A, TGFB2, CDK6, MLST8, INHBA, THBS1, UHMK1
Response to drug	0.002	10	CDKN1A, TGFB2, ACTC1, JUND, SEMA3C, CCND1, HMGCS1, MDK, INHBA, THBS1
Response to wounding	0.003	5	TGFB2, MDK, PDGFA, ADM, CTGF
Semaphorin-plexin signaling pathway	0.003	4	SEMA6B, SEMA3C, FLNA, MET
Sterol biosynthetic process	0.003	3	SQLE, MSMO1, LBR
Negative regulation of cell growth	0.005	6	GREM1, CDKN1A, TGFB2, GNG4, INHBA, ADIPOR1
Palate development	0.005	5	TGFBR3, TGFB2, ARID5B, INHBA, BMPR1A
Cardiac right ventricle morphogenesis	0.006	3	TGFB2, SEMA3C, BMPR1A
Regulation of cell growth	0.006	5	IGFBP3, CRIM1, SGK1, CYR61, CTGF
Positive regulation of cell migration	0.007	7	SEMA6B, EDN1, SEMA3C, PDGFA, CTSH, THBS1, CYR61
Isoprenoid biosynthetic process	0.007	3	HMGCS1, HMGCR, FDFT1
Negative regulation of smooth muscle cell migration	0.007	3	IGFBP3, SERPINE1, BMPR1A
Cell-cell signaling	0.008	8	GREM1, EDN1, TGFB2, PDGFA, ADM, INHBA, CYR61, CTGF
Regulation of G1/S transition of mitotic cell cycle	0.009	3	CCND1, PSME1, E2F1
Positive regulation of cell proliferation	0.010	11	GREM1, MYOCD, EDN1, TGFB2, PDGFA, CTSH, ADM, STX3, HIPK1, THBS1, CTGF

Positive regulation of fibroblast proliferation	0.013	4	CDKN1A, CDK6, E2F1, PDGFA
G1/S transition of mitotic cell cycle	0.014	5	CDKN1A, CDK6, CCND1, INHBA, SKP2
Vasculogenesis	0.014	4	MYOCD, CAV1, ADM, TEAD2
Regulation of glucose metabolic process	0.017	3	IGFBP3, ADIPOR1, PDK2
Negative regulation of peptidyl-serine phosphorylation	0.018	3	PDE4D, CAV1, PAQR3
Positive regulation of transforming growth factor beta receptor signaling pathway	0.020	3	TGFBR3, MYOCD, THBS1
Cell migration	0.020	6	TGFBR3, TGFB2, MDK, SDC3, THBS1, CTGF
Response to hypoxia	0.020	6	TGFBR3, MYOCD, TGFB2, CAV1, ADM, THBS1
Inactivation of MAPK activity	0.021	3	RGS4, DUSP5, CAV1
Actomyosin structure organization	0.025	3	FRMD6, ACTC1, EPB41
Liver development	0.030	4	TGFBR3, HMGCS1, GNPNAT1, ARID5B
Positive regulation of transcription from RNA polymerase II promoter	0.033	16	MYOCD, EDN1, JUND, SERPINE1, PIK3R2, INHBA, CBFA2T2, CYR61, KLF2, GREM1, PLSCR1, E2F1, NCOA7, MET, TEAD2, BMPR1A
Apoptotic process	0.034	11	GREM1, AVEN, PLSCR1, ACTC1, ARL6IP1, KCNIP3, IGFBP3, MX1, CTSH, SGK1, IER3
Blood vessel remodeling	0.034	3	TGFB2, SEMA3C, TGM2
Lateral mesoderm development	0.036	2	TEAD2, BMPR1A
Regulation of DNA biosynthetic process	0.036	2	CDKN1A, PDGFA
Positive regulation of transcription from RNA polymerase II promoter involved in myocardial precursor cell differentiation	0.036	2	GREM1, MYOCD
Lung alveolus development	0.038	3	ERRFI1, MYOCD, PDGFA
Epithelial to mesenchymal transition	0.038	3	TGFBR3, TGFB2, LIMS1
Negative chemotaxis	0.038	3	SEMA6B, SEMA3C, PDGFA
Positive regulation of cell differentiation	0.044	3	JUND, CYR61, CTGF
Response to purine-containing compound	0.044	2	HMGCS1, ADSS
Mesenchyme migration	0.044	2	ACTC1, ACTG2

Negative regulation of plasminogen activation	0.044	2	SERPINE1, THBS1
Hair follicle development	0.046	3	TGFB2, PDGFA, INHBA
Camp-mediated signaling	0.046	3	PDE4D, ADM, TGM2
Regulation of G-protein coupled receptor protein signaling pathway	0.048	3	RGS4, GNG4, HOMER2
Response to progesterone	0.048	3	TGFB2, CAV1, THBS1

Enrichment analysis based on all coding genes expressed by hMCs (mean channel intensity > 100, according to Clariom™ S Assay); GO BP: Gene Ontology Biological Processes; EASE (expression analysis systematic explorer) score < 0.05; $p < 0.05$

Supplementary Table S5:

Significantly enriched GO CC (direct) based on all DECGs

Term	P value	Count	Genes
Cytosol	$9.21*10^{-11}$	47	ERRFI1, CDKN1A, PDE1C, RBM8A, ARL6IP1, ARHGEF28, AP2A1, PIK3R2, ACTG2, CTGF, RGS4, PPP2CB, PRDX2, CCND1, CUX1, CALD1, NT5DC3, MLST8, GNPNAT1, FLNA, CTSH, SPDL1, SKP2, ADSS, TGM2, IER3, HMGCS1, PDE4D, KCNIP3, TPM1, MX1, ITPK1, SMC1A, COPZ1, ACLY, MOV10, PLSCR1, ACTC1, CDK6, DDAH1, OAS3, RHOJ, SMS, PSME1, SGK1, DCP2, LIMS1
Platelet alpha granule lumen	0.001	5	SRGN, TGFB2, SERPINE1, PDGFA, THBS1
Cyclin-dependent protein kinase holoenzyme complex	0.008	3	CDKN1A, CDK6, CCND1
Endoplasmic reticulum membrane	0.010	16	ARL6IP1, NUP210, CAV1, MX1, SSR3, MSMO1, HMGCR, ANTXR2, COPZ1, SQLE, LPCAT2, LRRC8C, TMEM106C, SGK1, MEST, FDFT1
Cortical cytoskeleton	0.016	3	EPB41, TRPC4, FLNA
Golgi membrane	0.017	12	SRGN, GOLT1B, CUX1, RNF24, MAN2A2, CAV1, LPCAT2, PAQR3, PDGFA, GNPNAT1, COPZ1, ST6GALNAC5
Extracellular matrix	0.017	8	TGFB2, PLSCR1, SERPINE1, TIMP3, FLNA, THBS1, CYR61, TGM2
Extracellular exosome	0.022	36	SEMA3C, SLC20A2, GSTCD, SERPINE1, NDUFB1, THBS1, DNPH1, ACTG2, PPP2CB, PRDX2, GNG4, GNA11, FLNA, TIMP3, CTSH, SH3BGRL2, CPNE2, FAM3C, STX3, TSPAN3, MEST, ADSS, TGM2, IGFBP3, CRIM1, TGFBR3, CLDN11, ACLY, PLSCR1, TMEM2, ACTC1, DDAH1, RAB15, RHOJ, SMS, PSME1

Enrichment analysis based on all coding genes expressed by hMCs (mean channel intensity > 100, according to Clariom™ S Assay); GO CC: Gene Ontology Cellular Compartment; EASE (expression analysis systematic explorer) score < 0.05; p < 0.05

Supplementary Table S6:

Significantly enriched GO MF (direct) based on all DECGs

Term	P value	Count	Genes
Insulin-like growth factor binding	$5.60*10^{-11}$	4	IGFBP3, CRIM1, CYR61, CTGF
Protein binding	9.63*10-9	105	ERRFI1, ARL6IP1, SERPINE1, WBP1L, ANTXR2, CTGF, CCND1, UBASH3B, KIF5C, CPNE2, FAM3C, SKP2, TGM2, IER3, IGFBP3, TPM1, UBE4A, ARID5B, UHMK1, ACLY, FRMD6, PSME1, RBM8A, EPB41, GSTCD, PDGFA, PIK3R2, GTF2E1, ATXN1, CALD1, BTBD2, COA3, TGFB2, JUND, HOMER2, CAV1, INHBA, MOV10, H1F0, CDK6, MXD4, BMPR1A, AVEN, CDKN1A, FHL2, ADM, AP2A1, DNPH1, ENC1, PBK, RBPMS2, MLST8, TIMP3, CTSH, SPDL1, LBR, TEAD2, CCDC50, SRGN, DUSP5, EDN1, MYOCD, TRPC4, GP1BB, SECISBP2L, PDE4D, SMC1A, CBFA2T2, C1QL1, TGFBR3, GREM1, PLSCR1, OAS3, RHOJ, TMEM106C, SLC25A10, NCOA7, SGK1, MET, DCP2, TAGLN, AMIGO2, HMGCR, SAMHD1, THBS1, PPP2CB, GNG4, LRRC8C, E2F1, FLNA, STX3, ADSS, VAT1L, ZNF460, RNF24, YTHDF3, KCNIP3, MX1, C1ORF21, HIPK1, KLF2, KIAA0101, RAB15, TACC1, LIMS1
3',5'-cyclic-nucleotide phosphodiesterase activity	0.019	3	PDE1C, PDE4D, UBE4A
Identical protein binding	0.021	14	ARL6IP1, GP1BB, CAV1, FHL2, INHBA, C1QL4, ADIPOR1, THBS1, DNPH1, CLDN11, ATXN1, UBASH3B, GNPNAT1, SKP2
Fibronectin binding	0.024	3	IGFBP3, THBS1, CTGF

Enrichment analysis based on all coding genes expressed by hMCs (mean channel intensity > 100, according to Clariom™ S Assay); GO MF: Gene Ontology Molecular Function; EASE (expression analysis systematic explorer) score < 0.05; $p < 0.05$

Supplementary Table S7:

Significantly enriched KEGG pathways based on all DECGs

**Term**	**P value**	**Count**	**Genes**
Melanoma	$1.04*10^{-12}$	7	CDKN1A, CDK6, CCND1, E2F1, PDGFA, PIK3R2, MET
p53 signaling pathway	$7.22*10^{-11}$	6	CDKN1A, CDK6, CCND1, IGFBP3, SERPINE1, THBS1
Glioma	$6.28*10^{-11}$	6	CDKN1A, CDK6, CCND1, E2F1, PDGFA, PIK3R2
FoxO signaling pathway	$5.53*10^{-11}$	8	CDKN1A, TGFB2, CCND1, HOMER2, PIK3R2, SKP2, SGK1, KLF2
Chronic myeloid leukemia	0.001	6	CDKN1A, TGFB2, CDK6, CCND1, E2F1, PIK3R2
Hippo signaling pathway	0.001	8	PPP2CB, TGFB2, FRMD6, CCND1, SERPINE1, TEAD2, BMPR1A, CTGF
Proteoglycans in cancer	0.001	9	CDKN1A, TGFB2, CCND1, CAV1, TIMP3, FLNA, PIK3R2, THBS1, MET
Cell cycle	0.002	7	CDKN1A, TGFB2, CDK6, CCND1, E2F1, SMC1A, SKP2
MicroRNAs in cancer	0.003	10	CDKN1A, TGFB2, CDK6, CCND1, TPM1, E2F1, PDGFA, TIMP3, THBS1, MET
PI3K-Akt signaling pathway	0.004	11	PPP2CB, CDKN1A, CDK6, CCND1, GNG4, MLST8, PDGFA, PIK3R2, SGK1, THBS1, MET
Pancreatic cancer	0.005	5	TGFB2, CDK6, CCND1, E2F1, PIK3R2
Pathways in cancer	0.009	11	CDKN1A, TGFB2, CDK6, CCND1, GNG4, GNA11, E2F1, PDGFA, PIK3R2, SKP2, MET
Bladder cancer	0.010	4	CDKN1A, CCND1, E2F1, THBS1
TGF-beta signaling pathway	0.012	5	PPP2CB, TGFB2, INHBA, THBS1, BMPR1A
Small cell lung cancer	0.013	5	CDK6, CCND1, E2F1, PIK3R2, SKP2
Prostate cancer	0.015	5	CDKN1A, CCND1, E2F1, PDGFA, PIK3R2
Steroid biosynthesis	0.019	3	SQLE, MSMO1, FDFT1
Hepatitis B	0.020	6	CDKN1A, TGFB2, CDK6, CCND1, E2F1, PIK3R2
Non-small cell lung cancer	0.022	4	CDK6, CCND1, E2F1, PIK3R2
Focal adhesion	0.023	7	CCND1, CAV1, PDGFA, FLNA, PIK3R2, THBS1, MET
Chagas disease (American trypanosomiasis)	0.025	5	PPP2CB, TGFB2, GNA11, SERPINE1, PIK3R2
AMPK signaling pathway	0.043	5	PPP2CB, CCND1, PIK3R2, HMGCR, ADIPOR1

Enrichment analysis based on all coding genes expressed by hMCs (mean channel intensity > 100, according to Clariom™ S Assay); KEGG: Kyoto Encyclopedia of Genes and Genomes; EASE (expression analysis systematic explorer) score < 0.05; p < 0.05

Supplementary Table S8:

Differentially expressed miRNAs after 24 h of silencing NEAT1

**Gene**	**x-fold change**	**P value**
hsa-miR-183-5p	2.46	0.0045
hsa-miR-339-5p	2.24	0.0009
hsa-miR-543	2.14	0.0021
hsa-miR-210-3p	1.95	0.0019
hsa-miR-199a-5p	1.93	0.0415
hsa-miR-370-3p	1.74	0.0115
hsa-miR-299-5p	1.71	0.0008
hsa-miR-615-3p	1.71	0.0160
hsa-miR-3180-3p	1.70	0.0304
hsa-miR-30c-5p	1.65	0.0133
hsa-miR-19b-3p	1.60	0.0338
hsa-miR-1307-3p	1.57	0.0139
hsa-miR-195-5p	1.57	0.0204
hsa-miR-6776-5p	-1.66	0.0118
hsa-mir-550a-1	-1.74	0.0147
hsa-mir-550a-2	-1.74	0.0147
hsa-mir-550a-3	-1.74	0.0147
hsa-miR-1247-3p	-1.99	0.0042
hsa-miR-92b-5p	-2.51	0.0001

Supplementary Table S9:

Differentially expressed miRNAs after 48 h of silencing NEAT1

**Gene**	**x-fold change**	**P value**
hsa-miR-652-3p	2.25	0.0271
hsa-miR-885-3p	2.18	0.0162
hsa-miR-126-3p	2.05	0.0235
hsa-miR-6848-5p	1.93	0.0224
hsa-miR-3180-3p	1.82	0.0090
hsa-miR-4728-5p	1.64	0.0296
hsa-miR-1343-5p	1.53	0.0362
hsa-miR-6806-3p	-1.52	0.0254
hsa-miR-92b-5p	-1.55	0.0351
hsa-miR-450b-5p	-1.84	0.0284
hsa-miR-548a-3p	-1.87	0.0155
hsa-miR-4663	-2.11	0.0091

Supplementary Table S10:

Significantly enriched GO BP based on DEmiRNAs

**Term**	**P value**	**Count of miRNAs**	**Count of genes**
Organelle	$1.22*10^{-4}$	18	67
Ion binding	0.0002	17	44
Cytosol	0.0003	15	28
Fibroblast growth factor receptor signaling pathway	0.0005	8	7
Cholesterol biosynthetic process	0.0046	4	4
Cellular lipid metabolic process	0.0046	4	5
Platelet degranulation	0.0046	5	4
Cyclin-dependent protein kinase holoenzyme complex	0.0046	8	3
Cell cycle arrest	0.0075	11	6
Cellular_component	0.0075	19	92
Mitotic cell cycle	0.0088	9	7
Platelet alpha granule lumen	0.0101	4	3
Response to drug	0.0115	8	9
Response to purine-containing compound	0.0183	3	2
Positive regulation of smooth muscle cell proliferation	0.0203	4	4
Fc-epsilon receptor signaling pathway	0.0203	4	4
Neurotrophin TRK receptor signaling pathway	0.0203	7	5
Epidermal growth factor receptor signaling pathway	0.0203	7	5
Negative regulation of phosphorylation	0.0203	8	3
G1/S transition of mitotic cell cycle	0.0203	9	5
Fibronectin binding	0.0253	3	3
Positive regulation of transforming growth factor beta receptor signaling pathway	0.0253	5	3
Cotranslational protein targeting to membrane	0.0286	2	2
Sec61 translocon complex	0.0322	2	2
Phosphatidylinositol-mediated signaling	0.0322	4	4
Type II transforming growth factor beta receptor binding	0.0363	3	2
Regulation of cell growth	0.0363	3	4
Negative regulation of epithelial cell proliferation	0.0363	8	4
Negative regulation of apoptotic process	0.0363	9	10
Cortical cytoskeleton	0.0370	4	3
Response to organonitrogen compound	0.0430	5	3
Response to organic cyclic compound	0.0430	6	5
Cellular response to follicle-stimulating hormone stimulus	0.0467	3	2
Epithelial to mesenchymal transition	0.0482	4	3

Supplementary Table S11:

Significantly enriched KEGG pathways based on DEmiRNAs

**Term**	**P value**	**Count of miRNAs**	**Count of genes**
Glioma	0.0012	10	5
p53 signaling pathway	0.0054	8	5
Viral carcinogenesis	0.0054	10	5
Melanoma	0.0054	10	5
Proteoglycans in cancer	0.0160	8	7
Non-small cell lung cancer	0.0160	8	3
Pathways in cancer	0.0160	11	7
FoxO signaling pathway	0.0172	8	7
Pancreatic cancer	0.0232	9	4
Cell cycle	0.0232	10	6
Chronic myeloid leukemia	0.0232	10	5
Small cell lung cancer	0.0246	9	4
TGF-beta signaling pathway	0.0435	8	5
PI3K-Akt signaling pathway	0.0461	12	8

Supplementary Table S12:

Identification of NEAT1 binding miRNAs using the three databases: RNA22, mirDIP, and Starbase

**RNA22**	**miRDIP**	**Starbase**
hsa-miR-339-5p	hsa-miR-339-5p	hsa-miR-339-5p
hsa-miR-615-3p	hsa-miR-615-3p	hsa-miR-615-3p
hsa-miR-3180y-3p	hsa-miR-3180y-3p	hsa-miR-3180y-3p
hsa-miR-885-3p	hsa-miR-183-5p	hsa-miR-183-5p
hsa-miR-92b-5p	hsa-miR-195-5p	hsa-miR-195-5p
hsa-miR-1343-5p	hsa-miR-299-5p	hsa-miR-299-5p
hsa-miR-4728-5p	hsa-miR-652-3p	hsa-miR-652-3p
hsa-miR-6776-5p	hsa-miR-885-3p	hsa-miR-19b-3p
hsa-miR-6848-5p	hsa-miR-126-3p	hsa-miR-30c-5p
	hsa-miR-210y-3p	hsa-miR-370y-3p
	hsa-miR-548a-3p	hsa-miR-450b-5p
	hsa-miR-1307-3p	hsa-miR-543

Supplementary table S12: miRNAs highlighted in yellow were identified by three databases, miRNAs highlighted in grey were identified by two databases. Non-color coded miRNAs were only identified by the respective database.

Supplementary Table S13:

Significantly enriched GAD diseases based on DECGs with potential miR-339-5p targeting site

Term	P value	Count	Genes
Type 2 diabetes, edema, rosiglitazone	$2.50*10^{-5}$	16	DUSP5, CDKN1A, EDN1, PDE4D, IGFBP3, SERPINE1, HMGCR, TGFBR3, RGS4, CDK6, NAV3, DDAH1, RAB15, FLNA, SKP2, LBR
Ovarian cancer	$2.50*10^{-4}$	7	DUSP5, CDKN1A, CDK6, OAS3, IGFBP3, SERPINE1, SKP2
Asthma	0.0050	6	TGFBR3, EDN1, LBH, NAV3, PDE4D, SERPINE1
Lung cancer	0.0080	6	CDKN1A, EDN1, RAB15, IGFBP3, SERPINE1, HMGCR
Bladder cancer	0.0090	6	CDKN1A, EDN1, CDK6, RAB15, IGFBP3, HMGCR
Sleep	0.0095	3	EPB41, PDE4D, ITPK1
Kidney dysfunction	0.0098	2	EDN1, SERPINE1
Atherosclerosis	0.0109	5	EDN1, PDE4D, IGFBP3, SERPINE1, HMGCR
Type 2 diabetes	0.0114	5	EDN1, DDAH1, IGFBP3, SERPINE1, HMGCR
Breast cancer	0.0147	6	CDKN1A, CDK6, IGFBP3, SERPINE1, TACC1, SKP2
Bone density	0.0173	4	TGFBR3, NAV3, PDE4D, SERPINE1
Obesity, premature ovarian failure	0.0233	4	TGFBR3, IGFBP3, SERPINE1, TACC1
Brain ischemia, stroke	0.0233	3	PDE4D, IGFBP3, SERPINE1
Thyroid diseases	0.0292	2	PDE4D, IGFBP3
Normal variation	0.0314	4	RGS4, EDN1, SERPINE1, HMGCR
Asthma	0.0341	3	EDN1, PDE4D, SERPINE1
Schizophrenia, schizoaffective disorder, bipolar disorder	0.0364	2	RGS4, ATXN1
Myocardial infarction	0.0415	5	ATXN1, NAV3, CUX1, SERPINE1, HMGCR
Stroke	0.0430	5	ATXN1, CUX1, PDE4D, SERPINE1, HMGCR
Mouth neoplasms, precancerous conditions	0.0435	2	CDKN1A, CDK6
Apoplexy, myocardial ischemia, stroke	0.0435	2	PDE4D, SERPINE1
Atherosclerosis, generalized	0.0458	2	EDN1, SERPINE1
Alzheimer's disease	0.0481	4	ATXN1, SERPINE1, ENTPD7, HMGCR
Overall effect	0.0482	2	CDKN1A, IGFBP3
Ovarian cancer	0.0494	3	CDKN1A, IGFBP3, SERPINE1

Supplementary Table S14:

Significantly enriched GO BP based on DECGs with potential miR-339-5p targeting site

**Term**	**P value**	**Count**	Genes
Positive regulation of smooth muscle cell proliferation	0.0069	3	EDN1, HMGCR, SKP2
Dephosphorylation	0.0138	3	DUSP5, NT5DC3, ITPK1
Regulation of receptor activity	0.0186	2	PDE4D, SERPINE1
G1/S transition of mitotic cell cycle	0.0191	3	CDKN1A, CDK6, SKP2
Platelet degranulation	0.0194	3	SRGN, SERPINE1, FLNA
Negative regulation of wound healing	0.0226	2	SERPINE1, HMGCR
Negative regulation of blood coagulation	0.0247	2	EDN1, SERPINE1
Negative regulation of smooth muscle cell migration	0.0288	2	IGFBP3, SERPINE1
Regulation of pH	0.0328	2	EDN1, PDK2
Regulation of glucose metabolic process	0.0449	2	IGFBP3, PDK2
Negative regulation of cell proliferation	0.0489	4	CDKN1A, CDK6, IGFBP3, MXD4

Supplementary Table S15:

Significantly enriched GO CC based on DECGs with potential miR-339-5p targeting site

**Term**	**P value**	**Count**	Genes
Cytosol	0.0171	14	CDKN1A, PDE4D, ITPK1, SMC1A, COPZ1, RGS4, CDK6, CUX1, DDAH1, OAS3, NT5DC3, FLNA, SKP2, DCP2
Cyclin-dependent protein kinase holoenzyme complex	0.0316	2	CDKN1A, CDK6
Cortical cytoskeleton	0.0461	2	EPB41, FLNA

Supplementary Table S16:

Chemicals, agents, kits, as well as software and devices with their manufacturers

**Chemicals, agents, kits**	**Manufacturers**
Attractene Transfection Reagent	Qiagen, Hilden, Germany
Accutase	Sigma Aldrich, Taufkirchen. Germany
Alexa Fluor 594 anti-wheat germ agglutinin (WGA) antibody	Invitrogen, Carlsbad, California, USA
BrdU Cell Proliferation Assay Kit	Biovision GmbH, Ilmenau, Germany
Clariom™ S Assay human	Thermo Fisher Scientific Inc., Waltham, Massachusetts, USA
Culture-Insert 2 Well in µ-Dish 35 mm	ibidi gmbH, Gräfelfing, Germany
Dimethyl sulfoxide (DMSO)	Sigma Aldrich, Taufkirchen. Germany
DNase I	Qiagen, Hilden, Germany
Dual-Glo® Luciferase Assay System	Promega, Fitchburg, Wisconsin, USA
Dulbecco's modified Eagle's medium (DMEM 21855)	Life Technologies, Carlsbad, California, USA
Fetal calf serum	Sigma Aldrich, Taufkirchen. Germany
Formaldehyd (37%)	Carl Roth GmbH, Karlsruhe, Germany
GeneChip™ miRNA 4.0 Array	Thermo Fisher Scientific Inc., Waltham, Massachusetts, USA
Glucose	Merck KGaA, Darmstadt, Germany
hsa-miR-339-5p miScript miRNA mimics or negative control	Qiagen, Hilden, Germany
Lipofectamin RNAiMAX	Thermo Fisher Scientific Inc., Waltham, Massachusetts, USA
Mannitol	AppliChem GmbH, Darmstadt, Germany
miCURY LNA miRNA PCR Assays	Qiagen, Hilden, Germany
miRCURY LNA RT Kit	Qiagen, Hilden, Germany
miRCURY LNA SYBR Green PCR Kit	Qiagen, Hilden, Germany
miRNeasy Mini Kit	Qiagen, Hilden, Germany
M-MLV Reverse Transkriptase	Promega, Fitchburg, Wisconsin, USA
NEBuilder HiFi DNA Assembly	New England Biolabs, Ipswich, Massachusetts, USA
NucleoSpin RNA Plus Kit	Machery-Nagel GmbH, Düren, Deutschland
Opti-MEM	Thermo Fisher Scientific Inc., Waltham, Massachusetts, USA
Penicillin	Life Technologies, Carlsbad, California, USA
PmeI restriction endonuclease	New England Biolabs, Ipswich, Massachusetts, USA
Primer (for sequences, see supplementary table S21)	Eurofins Genomics, Ebersberg Germany
pmirGLO Luciferase vector	Promega, Fitchburg, Wisconsin, USA
QuantiTect SYBR Green PCR Kit	Qiagen, Hilden, Germany
Random primers	Promega, Fitchburg, Wisconsin, USA
RNasin	Promega, Fitchburg, Wisconsin, USA
Single stranded DNA oligos for luciferase reporter gene vector	Eurofins Genomics, Ebersberg Germany

cloning (for sequences, see supplementary table 20)	
siPOOLs targeting NEAT1 or scrambled control	siTOOLs Biotech GmbH, Planegg, Germany
Streptomycin	Life Technologies, Carlsbad, California, USA
Thapsigargin (TG)	Tocris Bioscience, Bristol, United Kingdom
TGF-β	PeproTech GmbH, Hamburg, Germany
TNFa	PeproTech GmbH, Hamburg, Germany
Tunicamycin (TM)	Tocris Bioscience, Bristol, United Kingdom
ViewRNA™ ISH Cell Assay Kit	Thermo Fisher Scientific Inc., Waltham, Massachusetts, USA
Probe set for human NEAT1 (for ViewRNA™ ISH Cell Assay Kit)	Thermo Fisher Scientific Inc., Waltham, Massachusetts, USA

Supplementary Table S17:

Software and devices

**Software, devices**	**Manufacturers**
AxioStar Plus Observer Z1	Carl Zeiss AG, Jena, Germany
HistoQuest	TissueGnostics GmbH, Vienna, Austria
SPSS Statistics Version 21	IBM, New York, USA
NanoDrop 2000c spectrophotometer	PEQLAB Biotechnologie GmbH, Erlangen, Germany
Sigmaplot	Systat Software GmbH, Illinois,, USA
Tecan infinite 200 pro	Tecan Trading AG, Männedorf, Switzerland
Transcriptome Analysis Console (TAC) software 4.0	Thermo Fisher Scientific Inc., Waltham, Massachusetts, USA
ViiA 7 Real-Time PCR System	Thermo Fisher Scientific Inc., Waltham, Massachusetts, USA

Supplementary Table S18:

Data bases and online tools

**Database**	**Internet address**
Database for Annotation, Visualization, and Integrated Discovery (DAVID) v6.8	http://david.abcc.ncifcrf.gov/knowledgebase/
DIANA miRPath v.3.0	http://www.microrna.gr/miRPathv3
mirDIP	http://ophid.utoronto.ca/mirDIP/
Primer3	http://bioinfo.ut.ee/cgi-bin/primer3-0.4.0
RNA22	https://cm.jefferson.edu/rna22/
Starbase	http://starbase.sysu.edu.cn/index.php

Supplementary Table S19

Single stranded DNA oligo sequences for cloning of luciferase reporter gene vectors

**Target site**	**Forward primer (5`→ 3`)**
I) WT	5'homologSeq – CCAGGGCTTCAGGGGACAGACAGGGAT – 3'homologSeq
II) MUT	5'homologSeq – CACGCGATGACCGCACACTGTCCCAGT – 3'homologSeq
II) WT	5'homologSeq – TGTGATGCCATCTCACAGGCAGGGG – 3'homologSeq
III) MUT	5'homologSeq – ACACTTCGCAAGACACTCCGTCCCC – 3'homologSeq
III) WT	5'homologSeq – GGTGAGCCTGGGAGGGAGGGA – 3'homologSeq
III) MUT	5'homologSeq – GCACTCGGAGCCTCCCTCCCT – 3'homologSeq
IV) WT	5'homologSeq – CTTGAGCAAAGTGGGGGAGGGGGC – 3'homologSeq
IV) MUT	5'homologSeq – CTTCTCGAAAGACCTCCTGCCACC – 3'homologSeq

Structure of each single stranded DNA oligo
Homologous 5' sequence - NEAT1 WT or MUT target sequence - Homologous 3' sequence

Sequence of the homologous 5' sequence (5'homologSeq): GTGTAATTCTAGTTGTTTAAACGAGCT
Sequence of the homologous 3' sequence (3'homologSeq): CGCTAGCCTCGAGTCTAGAGTCGACCT
(both 5`→ 3`)

Supplementary Table S20

Primer sequences

Gene	Forward primer (5`→ 3`)	Reverse primer (5`→3`)
NEAT1_1/2	TTGCTTTGCTACAAGGTGGG	ATTTCCCCTGCCTGTGAGAT
NEAT1_2	ACAGTGGCAGGGTTCAATTC	CTCAGATGGGGAAATGGAGA
CCND1	CCCTCGGTGTCCTACTTCAA	AAGCGGTCCAGGTAGTTCAT
CDK6	GGATCTCTGGAGTGTTGGCT	AGGCCAGTCTTCTTCTCCTG
CDKN1A	TGTCTTGTACCCTTGTGCCT	AATCTGTCATGCTGGTCTGC
CTGF	GCCTATTCTGTCACTTCGGC	GTACACCGTACCACCGAAGA
E2F1	CTTCGTAGCATTGCAGACCC	AAAACATCGATCGGGCCTTG
GNG4	CCTCTCATCTGACGACTGACA	TGAGCTTCACAGTAGGCCAG
GREM1	TCTACATCCCCAGGCACATC	GTTCAGGGCAGTTGAGTGTG
IGFBP2	ACGAGTCTCAGAGCACAGAT	TCCATTTCTCTACGGCAGGG
PPIB	TGTGGTGTTTGGCAAAGTTC	GCTTCTCCACCTCGATCTTG
PDK2	ACATGGCTAAGCTCCTGTGT	CATGTGGTAGAGGTGGGAGG
TGFβ2	GACCCCACATCTCCTGCTAA	TAAAGTGGACGTAGGCAGCA
TGFBR3	TGAAGTGACTGGACGAGACG	CAGTTCACACAGTGCACCAG

Supplementary Figure S1

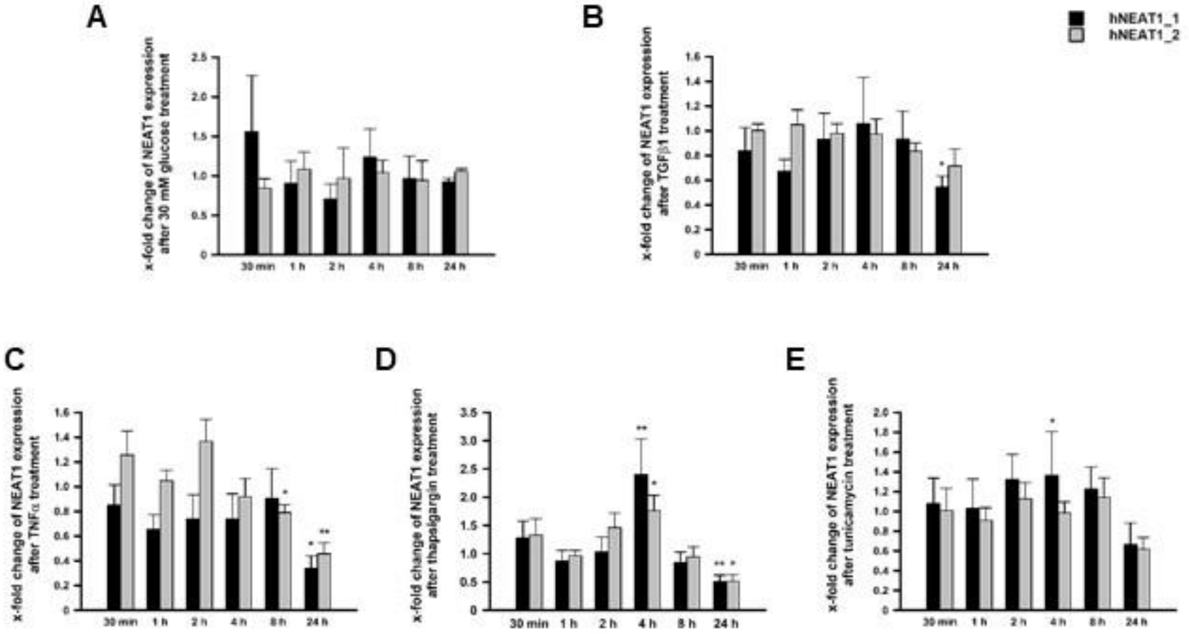

Supplementary Figure S1: NEAT1_1 and NEAT1_2 expression in human mesangial cell (hMCs) after stimulation. Change of NEAT1_1 (black bars) and NEAT1_2 (grey bars) RNA expression in hMCs after stimulation with A) 30 mM glucose normalized to mannitol, B) TGFβ1 normalized to medium, C) TNFα normalized to medium, D) thapsigargin normalized to DMSO, and E) tunicamycin normalized to DMSO. Bars represent x-fold change + SD. Overall significance of differences was analyzed by ANOVA, followed by a Student's t tests for post hoc pairwise comparisons. *) $p<0.05$; **) $p<0.001$ compared to corresponding control treatment; n= 3-4

Supplementary Figure S2

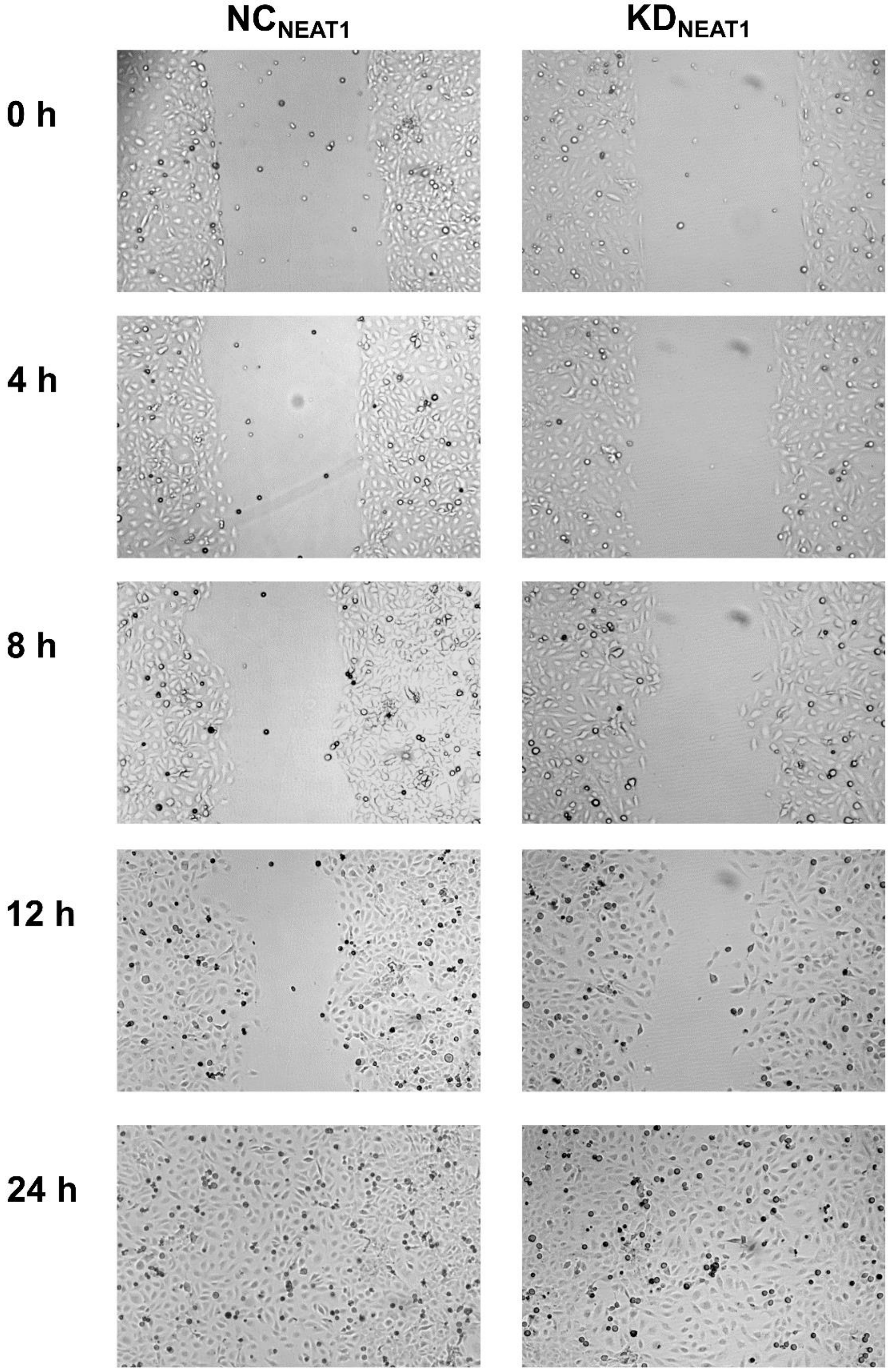

Supplementary Figure S2: Migration assay after KD_{NEAT1} in HMCs compared to cell treated with scrambled control siPools (NC_{NEAT1}) directly after creating the gap (0 h) as well as 4 h, 8 h, 12 h, and 24 h later.

Supplementary Figure S3

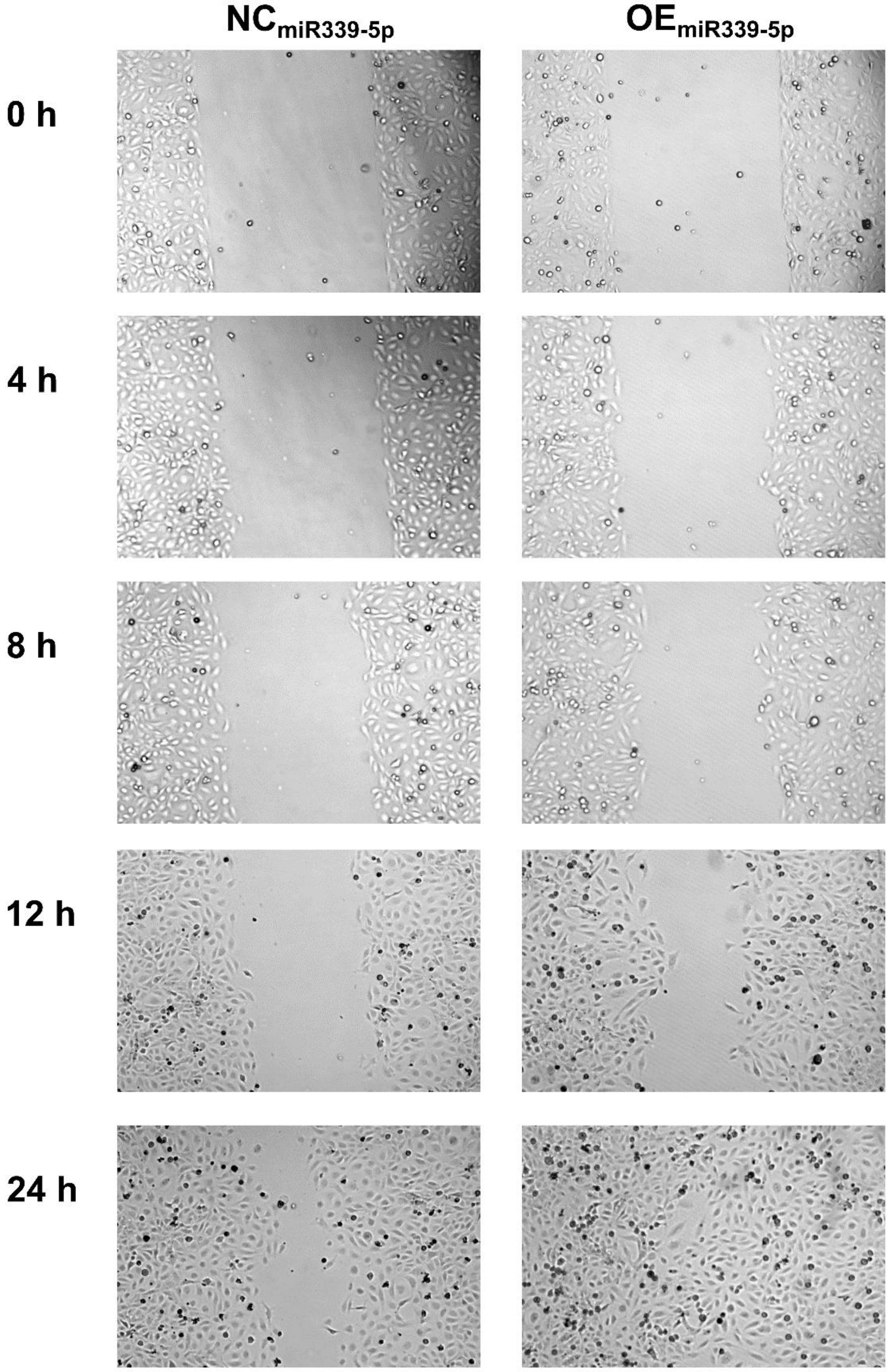

Supplementary Figure S3: Migration assay after $OE_{miR339\text{-}5p}$ in HMCs compared to cell treated with scrambled control mimcs ($NC_{miR339\text{-}5p}$) directly after creating the gap (0 h) as well as 4 h, 8 h, 12 h, and 24 h later.
